# Common profiles of Notch signaling differentiate disease-free survival in luminal type A and triple negative breast cancer

**DOI:** 10.18632/oncotarget.13451

**Published:** 2016-11-19

**Authors:** Magdalena Orzechowska, Dorota Jędroszka, Andrzej K Bednarek

**Affiliations:** ^1^ Department of Molecular Carcinogenesis, Medical University of Lodz, Lodz, 90-752, Poland

**Keywords:** Notch, breast neoplasms, epithelial-to-mesenchymal transition, disease-free survival

## Abstract

Breast cancer (BC) is characterized by high heterogeneity regarding its biology and clinical characteristics. The Notch pathway regulates such processes as organ modeling and epithelial-to-mesenchymal transition (EMT).

The aim of the study was to determine the effect of differential expression of Notch members on disease-free survival (DFS) in luminal type A (lumA) and triple negative (TN) BC.

The differential expression of 19 Notch members was examined in a TCGA BC cohort. DFS analysis was performed using the log-rank test (p<0.05). Biological differences between DFS groups were determined with Gene Set Enrichment Analysis (GSEA) (tTest, FDR<0.25). Common expression profiles according to Notch signaling were examined using ExpressCluster (K-means, mean centered, Euclidean distance metric).

The overexpression of *HES1, LFNG* and *PSEN1* was found to be favorable for DFS in lumA, and lowered expression favorable for DFS in TN.

GSEA analysis showed that differential Notch signaling is associated with cell cycle, tissue architecture and remodeling. Particularly, targets of E2F, early stage S phase transcription factor, were upregulated in the lumA unfavorable group and the TN favorable group differentiated on a basis of *HES1* and *PSEN1* expression.

Summarizing, our analysis show significance of Notch signaling in BRCA progression through triggering EMT. Moreover, identification of numerous genes which overexpression is associated with disease recurrence may serve as a source of potential targets for a new anticancer therapy.

## INTRODUCTION

Breast cancer (BC) is the most common tumor causing high mortality among women worldwide. It is characterized by high heterogeneity in respect of prognosis, clinical course, phenotype and molecular characteristics. A molecular classification of BC based on microarray studies has distinguished at least five subtypes with luminal type A (lumA), and a basal-like type comprising a triple negative immunophenotype (TN) [1–6]. While lumA BC is associated with a very good prognosis, the rapidly developing and metastatic TN BC has a poor clinical outcome.

The Notch pathway is an evolutionary conserved signaling mechanism determining cell fate and involved in regulation of proliferation, differentiation, vascular remodeling and angiogenesis in embryonic and adult tissues [7]. Mammals express four Notch receptors (*NOTCH1-NOTCH4*) and five DSL ligands: *DLL1, DLL3, DLL4, JAG1* and *JAG2*. The canonical Notch pathway is activated by interaction of DSL ligands with Notch receptors leading to two sequential proteolytic cleavages of the receptors: the first performed by ADAM/TACE metalloprotease, and the second in the remaining portion of Notch by the γ-secretase complex (comprising PSEN1, PSEN2, PEN2, APH1, nicastrin). This results in the release of the Notch intracellular domain (NICD), which in the nucleus forms a complex with DNA binding protein RPBJ and MAML family transcriptional coactivators. The latter induces the expression of Notch target genes encoding transcription factors (TFs), i.e. *HES1* and *HEY1* [8, 9].

Studies show that aberrant Notch signaling plays an important role in breast cancer development and progression via promotion of growth, invasion, angiogenesis and metastasis. Interestingly, the Notch pathway can be either tumor suppressive or oncogenic, depending on the expression profiles of its receptors and ligands [10]. In particular, high expression of *NOTCH1* and *NOTCH3* has been reported to be associated with hormone receptor-negative tumors. *NOTCH4*, similar to *NOTCH1* and *NOTCH3* functions as an oncogene, but with hormone receptor-positive BC [11]. On the contrary, high expression of *NOTCH2* has been associated with better survival [12], which might be associated with increased apoptosis *in vitro* [13]. Other studies indicate that elevated *NOTCH1* and *JAG1* in patients with BC is correlated with poor overall survival [14], and that the loss of *NUMB* expression increases Notch activity and thus increases proliferation of tumor cells [15].

Epithelial-to-mesenchymal transition (EMT) is a key mechanism for differentiating cells in complex tissues [16]. During tumor progression, various processes associated with EMT may increase the motility and invasiveness of cancer cells. When they become mesenchymal stem cells, epithelial cells lose their polarity, adherens junctions, tight junctions and cytokeratin intermediate filaments, but gain migratory properties [17, 18]. These changes can occur concomitantly with the upregulation of *SNAIL*, *SIP1/ZEB2* and *SLUG*, which are the direct transcriptional repressors of E-cadherin, and also with the acquisition of mesenchymal markers such as vimentin, N-cadherin and fibronectin [18, 19]. Among the many mechanisms implicated in EMT progression, accumulating evidence shows that the Notch signaling pathway is important in many human malignancies. *NOTCH1* has been found to be the major regulator of invasion and metastasis in esophageal carcinoma by inducing EMT through *SNAIL in vitro* [20]. In colorectal HT29 cancer cells, elevated expression of the Notch intracellular domain (NICD) and NFkβp65 resulted in the upregulation of *BCL-XL*, which subsequently led to inhibition of apoptosis and greater tumor progression [21]. Furthermore, the activation of *NOTCH1* was crucial for TGF β-induced EMT in epithelial ovarian cancer and was manifested by inhibition of E-cadherin [22].

The present study examines whether the differential expression of Notch signaling members has any effect on disease-free survival (DFS) in lumA and TN BC. We focused on luminal type A and triple negative breast cancers as the most biologically distant subtypes of breast cancer. Moreover, they are characterized by completely different hormone receptor status (ER+, PR+, HER2- vs ER-, PR-, HER2-), which is considered as cellular proliferation and differentiation factor itself that contributes to distinct characteristics of both cancer types. It attempts to identify the common and unique expression profiles of Notch targets differentiating lumA and TN BC, which may be potentially considered as prognostic biomarkers. Our results indicate that the altered expression of particular Notch signaling genes may play a role in the activation of EMT related processes and affect tissue architecture and remodeling.

## RESULTS

### Disease free survival analysis

**Figure 1:**
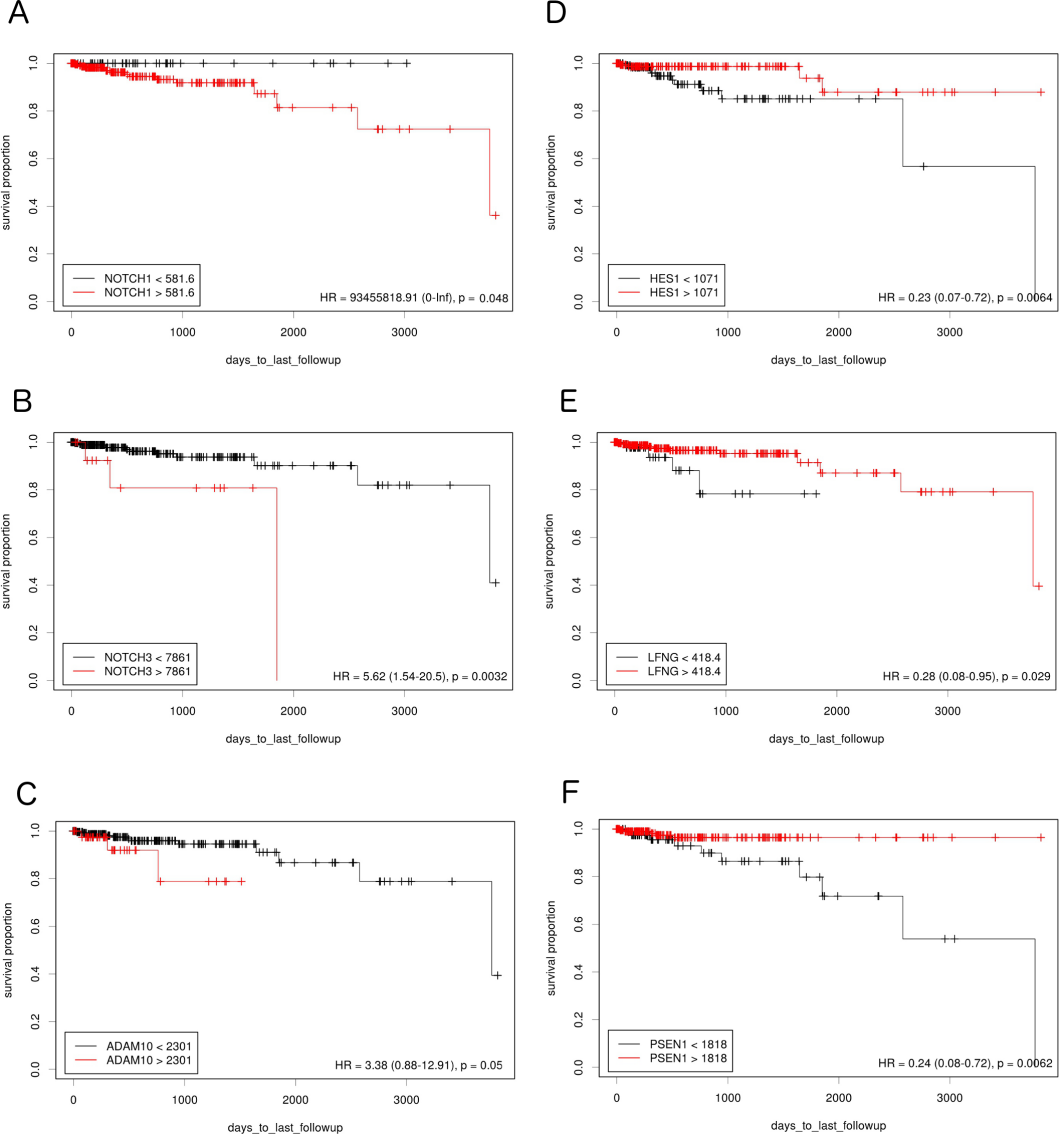
The prognostic effect of Notch member expression on DFS in lumA BC Kaplan-Meier curves are plotted for **A**. *NOTCH1*, **B**. *NOTCH 3*, **C**. *ADAM10*, **D**. *HES1*, **E**. *LFNG* and **F**. *PSEN1*.

**Figure 2:**
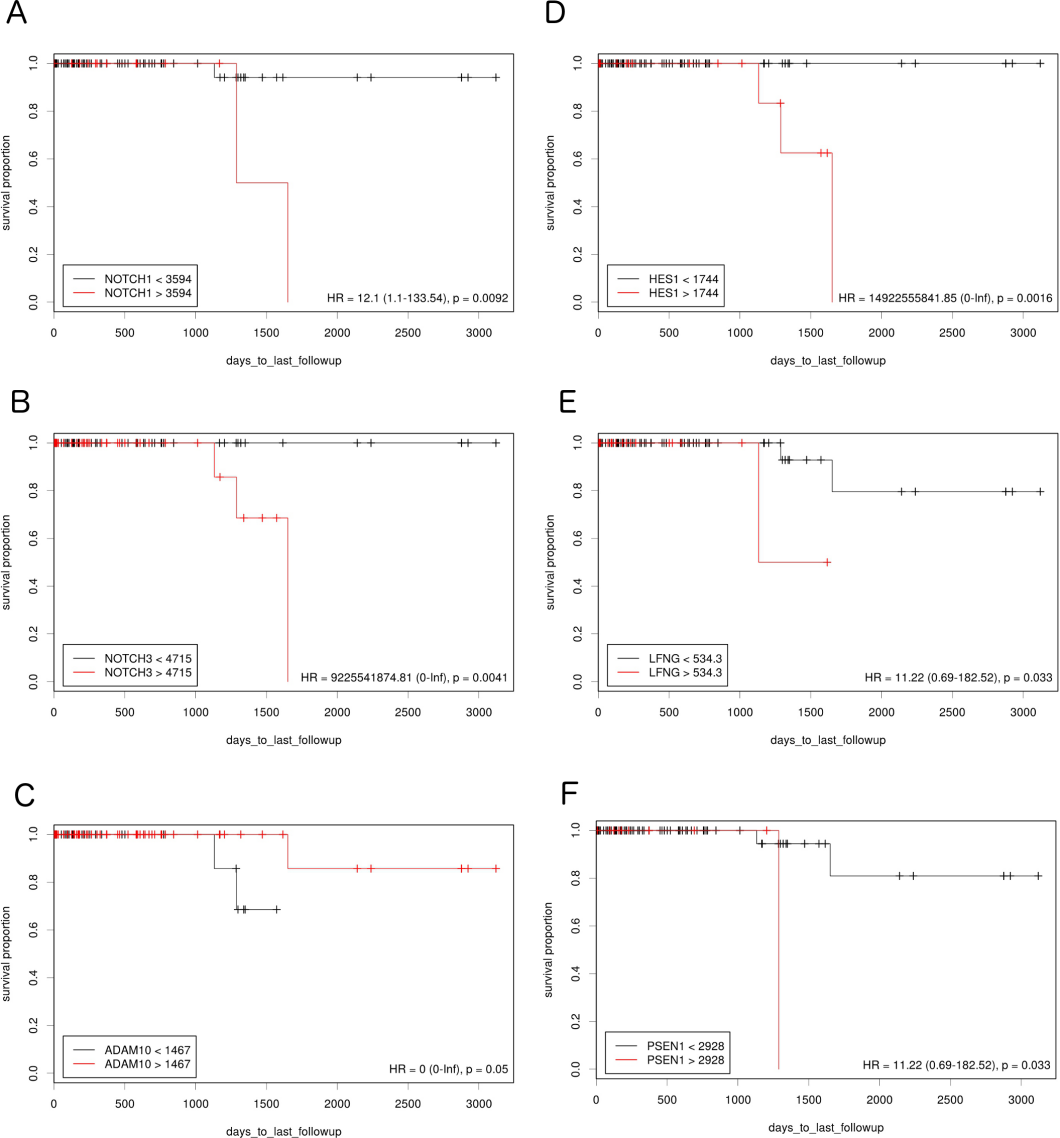
The prognostic effect of Notch member expression on DFS in TN BC Kaplan-Meier curves are plotted for A. *NOTCH1*, **B**. *NOTCH 3*, **C**. *ADAM10*, **D**. *HES1*, **E**. *LFNG* and **F**. *PSEN1*.

The study examined how differences in Notch pathway gene expression influence DFS in lumA and TN BC. Expression cutoff points and the numbers of patients assigned to groups based on low or high Notch gene expression are listed in Table 1. Differential expression of several genes like *APH1B, DLK1, JAG1, NOTCH4, PSEN2, HES5* had no significant effect on DFS, and were therefore excluded from further analyses. Remaining members of Notch signaling demonstrated contrary effect on DFS in both breast cancer subtypes. Specifically, relatively high expression of *HES1, PSEN1* and *LFNG* was correlated with good prognosis in lumA (HR=0.23, p=0.0064; HR=0.24, p=0.0062; HR=0.28, p=0.029, respectively) (Figure 1), while lowered expression was associated with better DFS in TN (HR>100, p=0.0016; HR=11.22, p=0.033; HR=11.22, p=0.033, respectively) (Figure 2).

**Table 1:** Statistics for DFS analysis

Gene	Cutoff		Number of patients in group	
			Low expression*	High expression*
		lumA BC		
*ADAM10*	2301		304	63
*ADAM17*	577.3		133	234
*APH1B*	710.3		237	130
*HES1*	1071		159	208
*HES4*	115.7		269	98
*HEY1*	558.3		350	17
*LFNG*	418.4		57	310
*NOTCH1*	581.6		67	300
*NOTCH3*	7861		348	19
*NUMB*	1738		271	96
*PSEN1*	1818		99	268
		*TN BC*		
*ADAM10*	1467		34	80
*HES1*	1744		95	19
*LFNG*	534.3		90	24
*NOTCH1*	3594		96	18
*NOTCH2*	7515		85	29
*NOTCH3*	4715		69	45
*PSEN1*	2928		98	16

*We defined “low expression” as the expression values below and “high expression” as the expression values above the determined cutoff.

On the contrary, lowered expression of *ADAM10* was correlated with better prognosis in lumA (HR=3.38, p=0.05) (Figure 1) and higher expression with better prognosis in TN (HR<0.001, p=0.05) (Figure 2). Lowered *NOTCH1* and *NOTCH3* expression was favorable for DFS in both lumA (HR>100, p=0.048; HR=3.91, p=0.023, respectively) (Figure 1) and TN (HR=12.1, p=0.0092; HR=100, p=0.0041, respectively) (Figure 2).

Some of the analyzed genes demonstrated a significant impact in only one of the subtypes. In particular, lowered expression of *NOTCH2* was correlated with better prognosis in TN (HR=100, p<0.001) and *ADAM17*, and *HEY1* in lumA (HR=4.71, p=0.011; HR=4.42, p=0.012, respectively); in contrast, overexpression of *DLL4, JAG2* and *NUMB* was favorable in lumA (HR=0.29, p=0.044; HR=0.3, p=0.023; HR=0, p=0.025, respectively).

### Gene enrichment analysis of Notch pathway downstream effect

#### Transcription factor binding motifs

**Figure 3:**
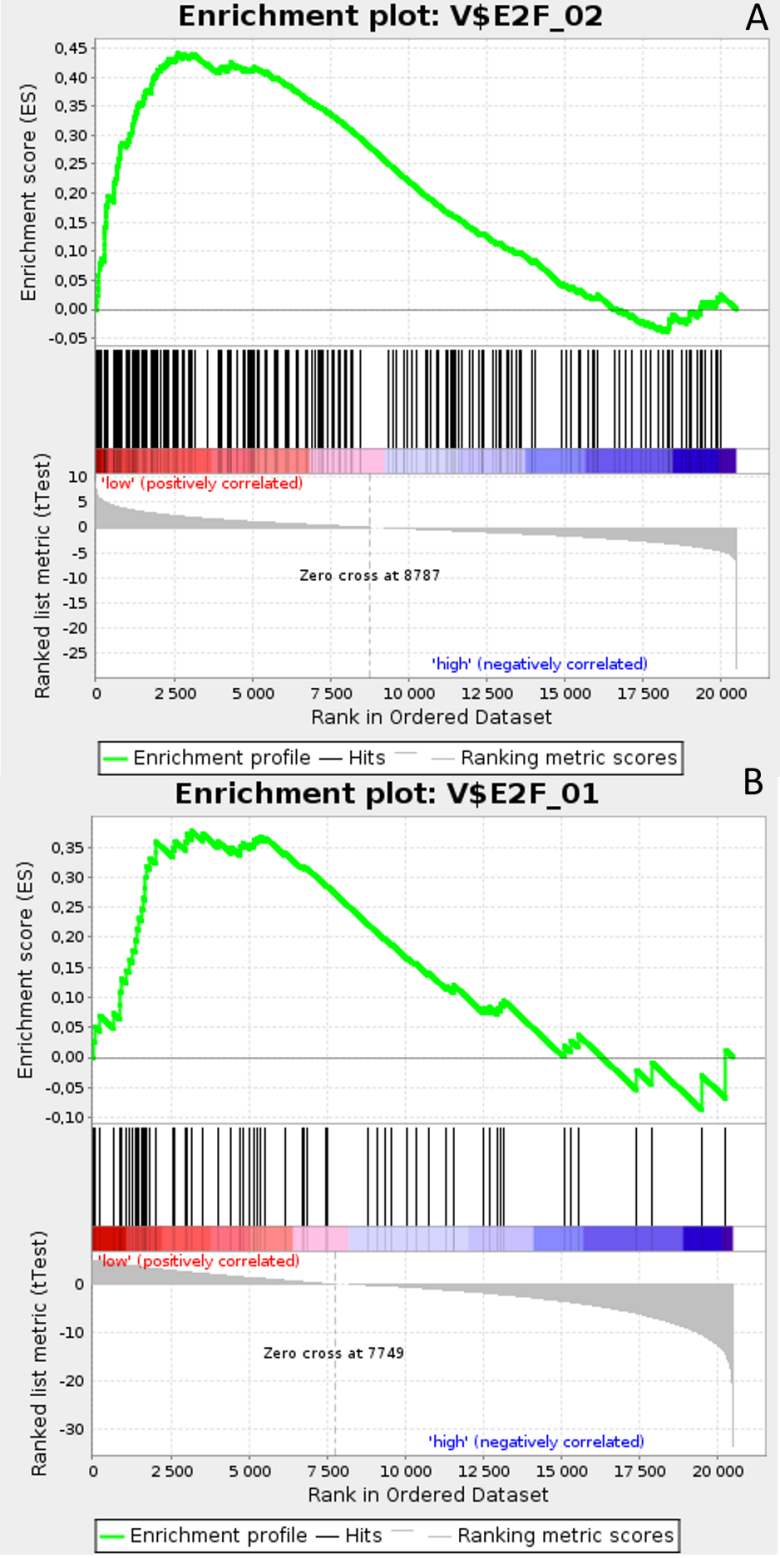
Enrichment plots presenting E2F target gene set in lumA **A**. *HES1*-low subgroup, **B**. *PSEN1*-low subgroup.

**Figure 4:**
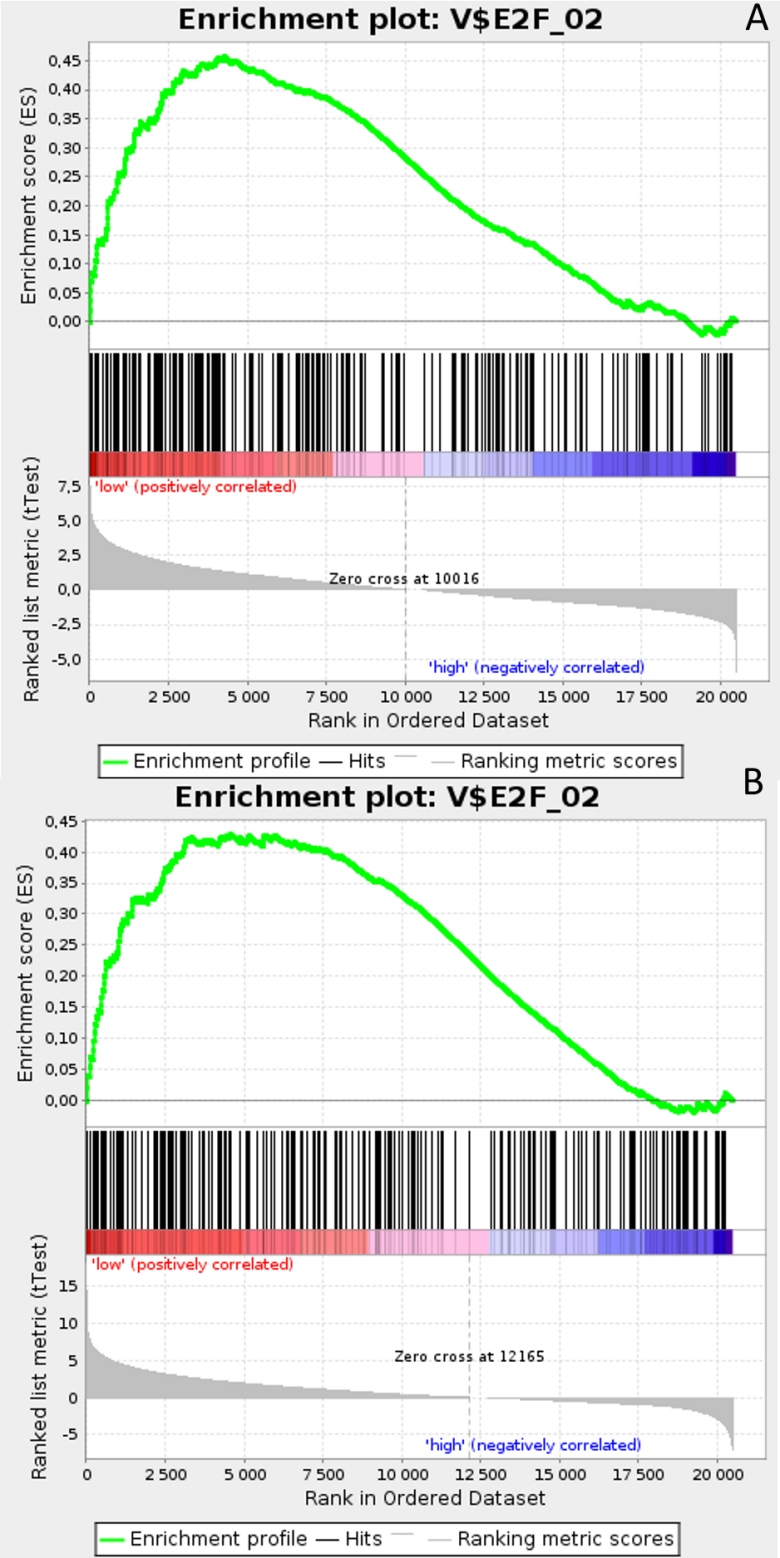
Enrichment plots presenting E2F target gene set in TN **A**. *HES1*-low, **B**. *PSEN1*-low subgroups.

**Figure 5:**
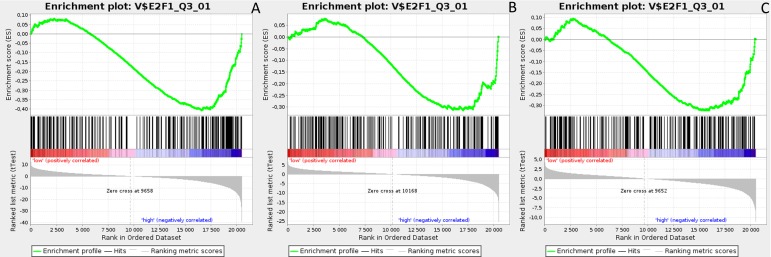
Enrichment plots presenting E2F1 targets in lumA **A**. *ADAM10*-high, **B**. *NOTCH1*-high, **C**. *NOTCH3*-high subgroups.

**Figure 6:**
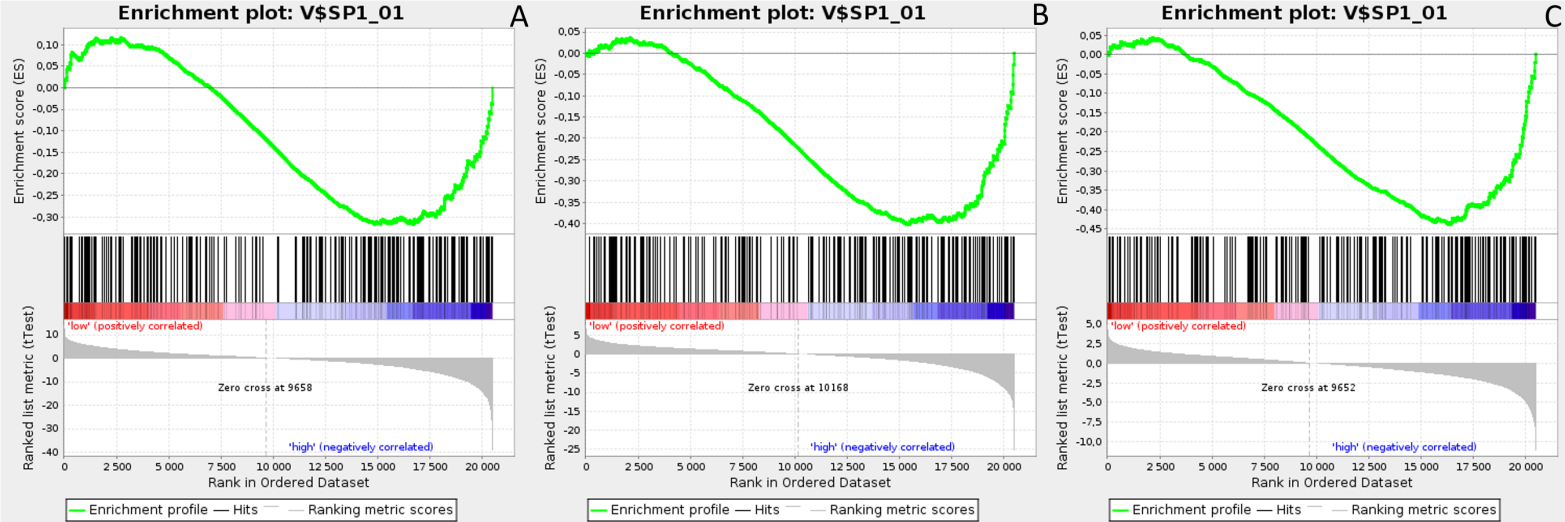
Enrichment plots presenting SP1 targets in lumA **A**. *ADAM10*-high, **B**. *NOTCH1*-high, **C**. *NOTCH3*-high subgroups.

**Figure 7:**
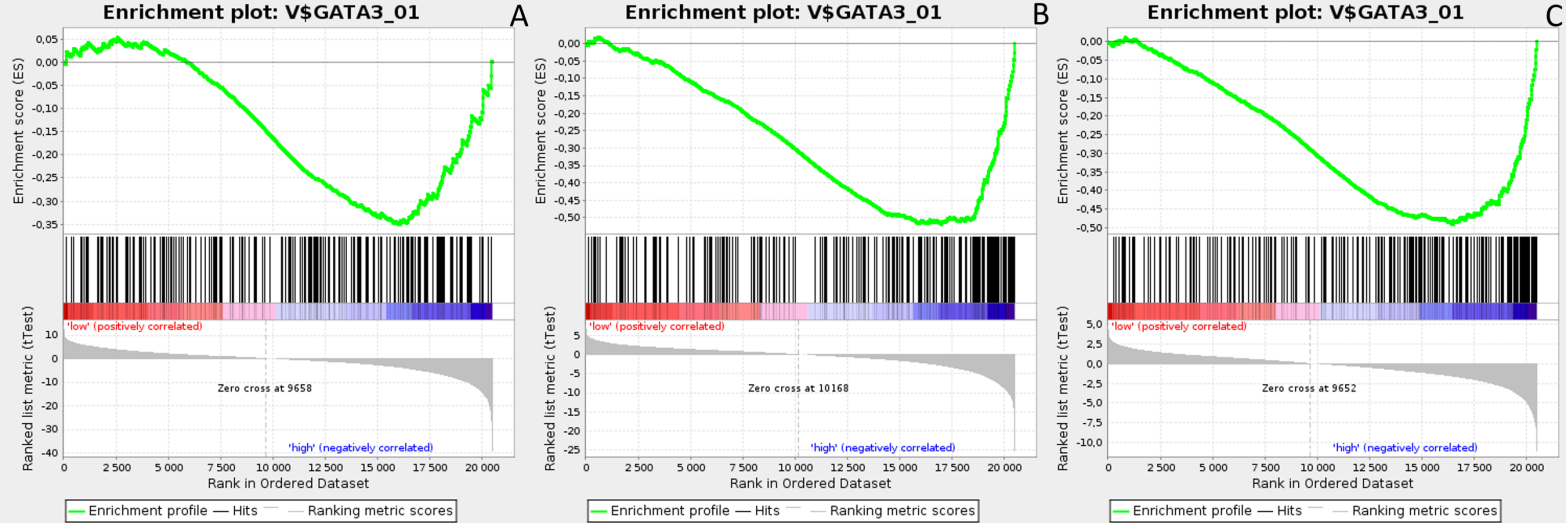
Enrichment plots presenting GATA3 targets in lumA **A**. *ADAM10*-high, **B**. *NOTCH1*-high, **C**. *NOTCH3*-high subgroups.

Gene Set Enrichment Analysis (GSEA) was performed to examine global biological differences between DFS groups based on previously computed cutoff points for Notch pathway genes for which differential expression had a significant influence on disease recurrence predicted outcome. GSEA of the molecular signatures of TF binding motifs found distinct associations between Notch signaling and TF involved in the regulation of cell cycle, tissue architecture and remodeling. In particular, targets of the E2F TF family were upregulated in *HES1* and *PSEN1* lumA DFS bad prognosis group (Figure 3) and TN good prognosis group (Figure 4), as well as in the *NOTCH1* bad prognosis group in both subtypes. *E2F1* targets were significantly upregulated in the lumA *ADAM10*, *NOTCH1* and *NOTCH3* bad prognosis phenotypes (Figure 5). Similar results were found for *SP1* target genes (Figure 6). In addition, *SP1* was found to be upregulated in the *ADAM10* lumA high phenotype (ES>0.1), which indicated that its upregulation contributes to a lumA-favorable prognosis, although at a considerably lower level (Figure 6). Interestingly, *SP1* different targets were upregulated in bad and good prognosis groups of lumA according to *ADAM10* differentiation. Furthermore, *GATA3* target genes were upregulated in the poorer prognosis groups of lumA *ADAM10*, *NOTCH1* and *NOTCH3* (Figure 7), while no significant upregulation in *GATA3* targets was found in the TN subtype. Additionally, targets of *LEF1*, *AP1*, *SRF*, *SMAD* and *NFKB* were significantly upregulated in unfavorable prognosis lumA *NOTCH1* and *NOTCH3*. Statistics for upregulated TF gene sets are listed in Table 2. Detailed results are available as Supplementary File 1.

**Table 2:** Selected TF targets gene sets for *ADAM10*, *NOTCH1* and *NOTCH3* unfavorable prognosis in lumA

Transcription factors	*ADAM10*		*NOTCH1*		*NOTCH3*	
	FDR	p-value	FDR	p-value	FDR	p-value
E2F_Q2	0.015	0.022	0.156	0.186	0.493	0.463
E2F1_Q3_01	0.012	0<0.001	0.094	0.053	0.094	0.039
SP1_Q6	0.047	0.002	0.005	0<0.001	0.140	0.079
GATA3	0.225	0.197	0<0.001	0<0.001	0.003	0<0.001
AP1_Q2	0.132	0.071	0<0.001	0<0.001	0.012	0.002
SMAD_Q6	0.012	0.001	0<0.001	0<0.001	0.007	0<0.001
SRF_Q6	0.155	0.178	0<0.001	0<0.001	0<0.001	0<0.001
NFKB_Q6	0.144	0.134	0<0.001	0<0.001	0.007	0<0.001
P53_02	0.043	0.008	0<0.001	0<0.001	0.005	0<0.001
LEF1_Q2	0.008	0<0.001	0.003	0.001	0.010	0.004
HIF1_Q3	0.001	0<0.001	0.039	0.010	0.246	0.192
MYC_Q2	0.204	0.180	0.201	0.202	0.288	0.268

### GSEA analysis of gene ontology (BP, CC, MF), KEGG canonical pathways and chemical and genetic perturbations

Results varied between lumA/TN and favorable/unfavorable phenotypes (determined by DFS analysis) according to differential expression of the Notch signaling pathway; however, only the significantly upregulated gene sets related to EMT were analyzed. Specifically, only gene sets containing the genes *VIM*, *MMP2*, *CDH2*, *ITGA5*, *ITGB6*, *SPARC*, *FN1* and *VNT* were significantly upregulated in *ADAM10*, *NOTCH1* and *NOTCH3* unfavorable prognosis (Table 3). Detailed results are available as Supplementary File 2.

**Table 3:** Selected gene sets regarding GO BP, CC, MF and KEGG canonical pathways for *ADAM10, NOTCH1* and *NOTCH3* unfavorable prognosis in lumA, essential in EMT

Hallmark gene set	EMT genes	*ADAM10*	*NOTCH1*	*NOTCH3*
GO Biological process				
Tissue remodeling	*SPARC*	-	0.003	0.019
Tissue development	*SMAD2, SPARC*	-	0.002	0.014
Transmembrane receptor protein tyrosine kinase signaling pathway	*FOXC2, SMAD2*	0.139	0.003	0.033
TGF- β receptor signaling pathway	*SMAD2, SMAD3*	0.035	0.003	0.058
GO Cellular compartment				
Extracellular region	*MMP2, MMP3, MMP9, VNT*	-	0.002	0.004
Cytoskeleton	*VIM, CTNNB1*	0.058	0.025	0.031
Integrin complex	*ITGA5, ITGB6*	0.041	0.01	0.017
Receptor complex	*ITGA5, ITGB6, SMAD3*	0.111	0.006	0.041
GO Molecular function				
Structural molecule activity	*VIM*	-	0.06	0.068
Structural constituent of cytoskeleton	*VIM*	-	0.007	0.007
KEGG Canonical pathway				
Adherens junction	*SMAD2, SMAD3, CTNNB1*	0.001	0.002	0.018
Tight junction	*CTNNB1*	0.158	0.002	0.016
Focal adhesion	*ITGA5, ITGB6, FN1, CTNNB1, VTN*	0.034	<0.001	<0.001
ECM receptor interaction	*ITGA5, ITGB6, FN1, VTN*	0.056	<0.001	0.002
Regulation of actin cytoskeleton	*ITGA5, ITGB6, FN1*	0.029	<0.001	0.019
Cell adhesion molecules cams	*CDH2*	-	<0.001	0.21
TGF- β signaling pathway	*SMAD2, SMAD3*	0.005	<0.001	0.015
Wnt signaling pathway	*CTNNB1, SMAD2, SMAD3*	0.041	0.003	0.049

GSEA of chemical and genetic perturbations (CGPs) indicated the upregulation of gene sets associated with resistance/sensitivity to various treatment and aberrant processes related to cancer progression/metastasis. *ADAM10*, *NOTCH1* and *NOTCH3* unfavorable prognosis groups demonstrated similar profiles of response to treatment, showing upregulation in resistance to doxorubicin, alkylating agents, endocrine therapy, mitoxantrone, dasatinib and cisplatin as well as sensitivity to fluorouracil, cyclophosphamide and vincristine. Additionally, upregulation was found in *CTNNB1* oncogenic signature, metastasis, EMT and metastasis through EMT (Table 4). Detailed results are available as Supplementary File 3.

**Table 4:** Selected CGPs in lumA unfavorable prognosis groups

	*ADAM10*	*HES1*	*NOTCH1*	*NOTCH3*	*PSEN1*
Doxorubicin resistance	0.108	-	0.001	0.007	-
Tamoxifen resistance dn	0.204	0.231	-	-	-
Alkylating agents resistance up	-	-	0.002	0.022	-
Alkylating agents resistance dn	0.034	0.193	-	-	0.221
Endocrine therapy resistance	0.001	-	0.25	0.156	-
Mitoxantrone resistance	0.006	-	0.033	0.108	-
Dasatinib resistance up	0.232	-	0.001	0.005	-
Cisplatin resistance up	0.245	-	0.005	0.008	-
Sensitivity to fluorouracil	0.019	-	0.023	0.191	-
Sensitivity to cyclophosphamide	0.065	-	-	-	-
Sensitivity to vincristine	0.231	-	0.045	0.021	-
*CTNNB1* oncogenic signature	0.000	0.15	0.082	0.214	-
Metastasis up	0.001	0.191	0.22	-	0.042
Epithelial-to-mesenchymal tansition up	0.22	-	0.000	0.000	-
Cancer mesenchymal transition signature	-	-	0.019	0.005	-
Metastasis EMT up	0.177	-	0.063	0.029	-

**Figure 8:**
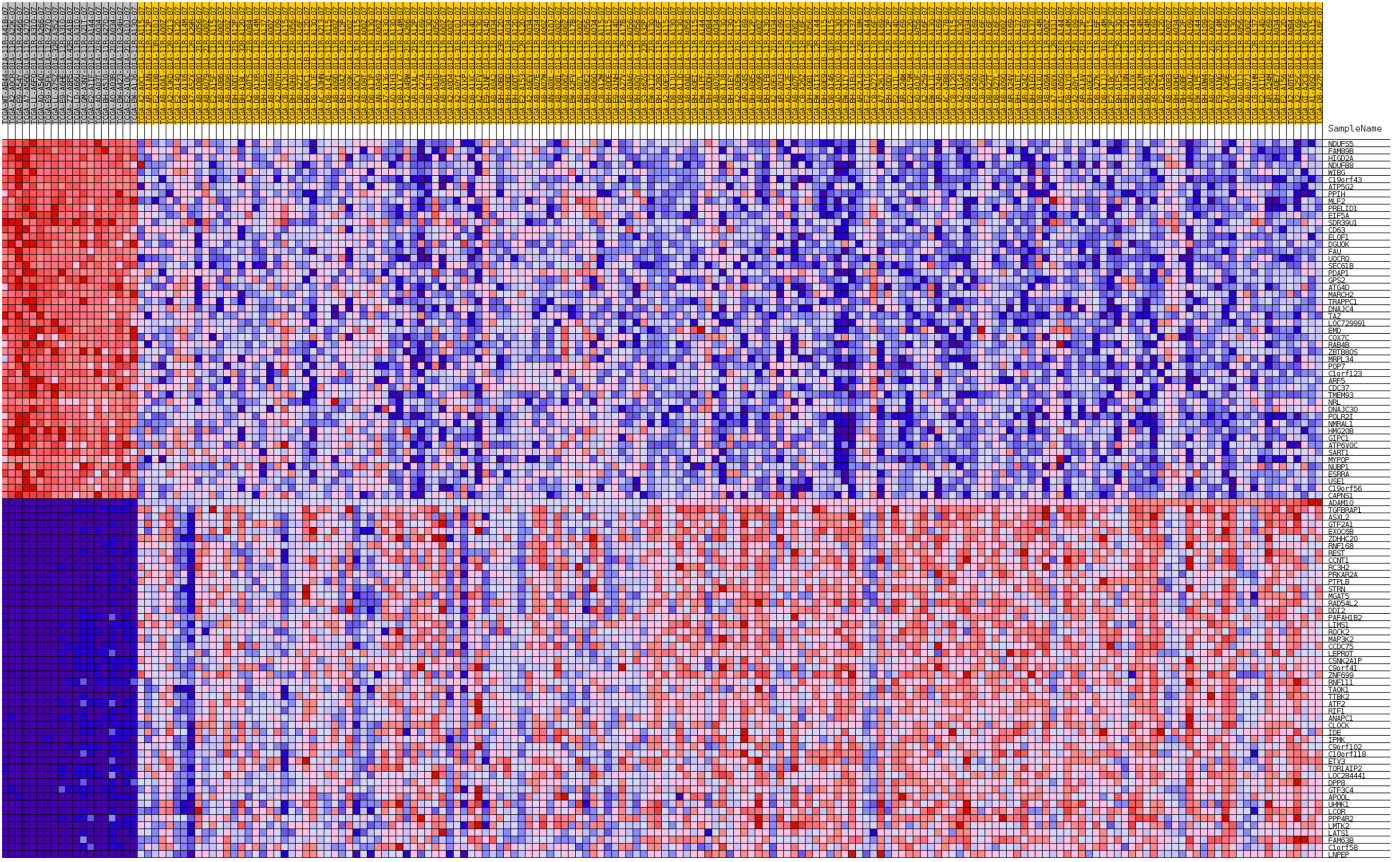
Heatmap of 50 marker genes for ADAM10 lumA phenotypes

**Figure 9:**
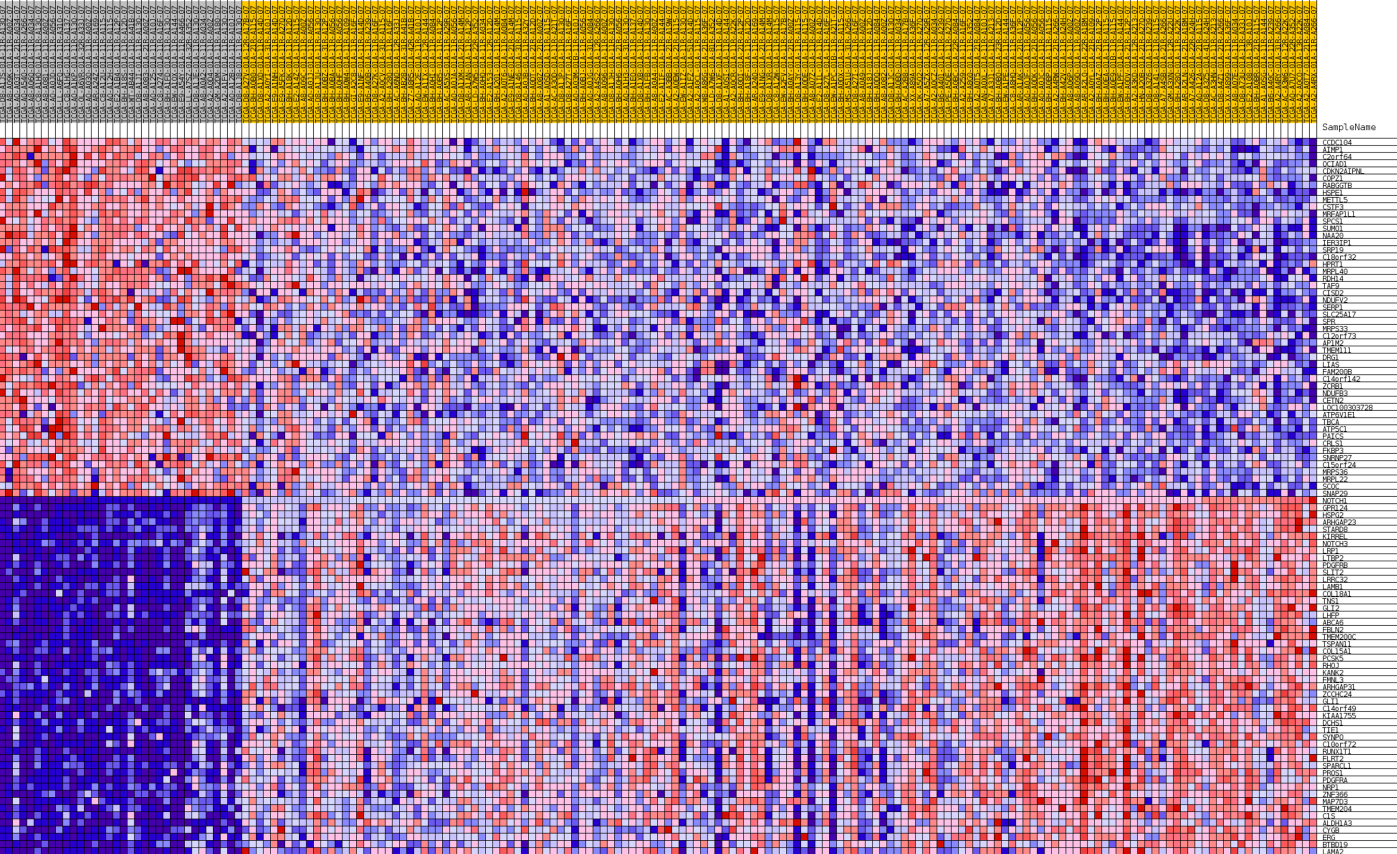
Heatmap of 50 marker genes for NOTCH1 lumA phenotypes

Additionally, GSEA heat maps were generated for the top 50 gene markers for each phenotype of DFS prognosis. Figures 8 and 9 present heat maps for the lumA *ADAM10* and *NOTCH1* phenotypes, showing the marker genes for comparing good and bad prognoses. Selected markers of *ADAM10* and *NOTCH1* unfavorable prognosis are listed in Table 5. Heatmaps of the gene markers for *HES1*, *PSEN1*, *LFNG* and *NOTCH3* phenotype are available as Supplementary Files 4-7.

**Table 5:** Marker genes for *ADAM10* and *NOTCH1* lumA phenotypes

*ADAM10*	*NOTCH1*
*ANAPC1, APOOL, ASXL2, ATF2, C10orf118, C1orf58, C9orf102, C9orf41, CCDC75, CCNT1, CLOCK, CSNK2A1P, DDI2, DPP8, ETV3, EXOC6B, FAM63B, GTF2A1, GTF3C4, IDE, IPMK, LATS1, LCOR, LEPROT, LIMS1, LMTK2, LNPEP, LOC284441, MAP3K2, MGAT5, PAFAH1B2, PPP4R2, PRKAR2A, PTPLB, RAD54L2, RC3H2, REST, RIF1, RNF111, RNF168, ROCK2, STRN, TAOK1, TGFBRAP1, TOR1AIP2, TTBK2, UHMK1, ZDHHC20, ZNF699*	*ABCA6, ALDH1A3, ARHGAP23, ARHGAP31, BTBD19, C10orf72, C14orf49, C1S, COL15A1, COL18A1, CYGB, DCHS1, ERG, FBLN2, FLRT2, FMNL3, GLI1, GLI2, GPR124, HSPG2, KANK2, KIAA1755, KIRREL, LAMA2, LAMB1, LHFP, LRP1, LRRC32, LTBP2, MAP7D3, NOTCH3, NRP1, PCSK5, PDGFRA, PDGFRB, PROS1, RHOJ, RUNX1T1, SLIT2, SPARCL1, STARD8, SYNPO, TIE1, TMEM200C, TMEM204, TNS1, TSPAN11, ZCCHC24, ZNF366*

### LumA and TN BC gene expression profiles comparison (cluster and class analysis)

**Figure 10:**
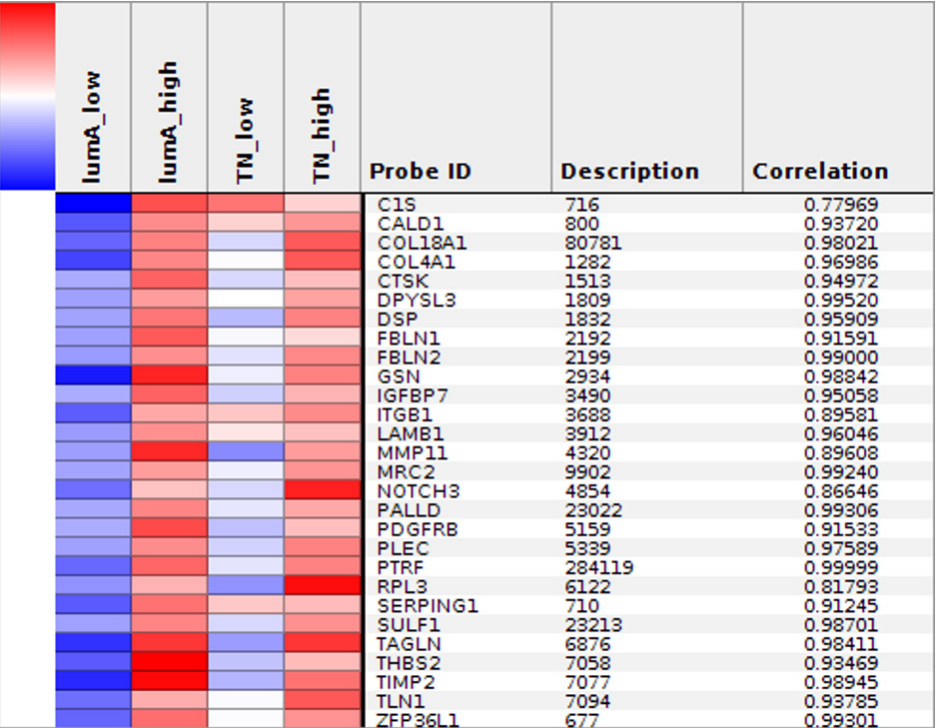
Heatmap representing common profiles in NOTCH1 lumA/TN unfavorable phenotypes

**Figure 11:**
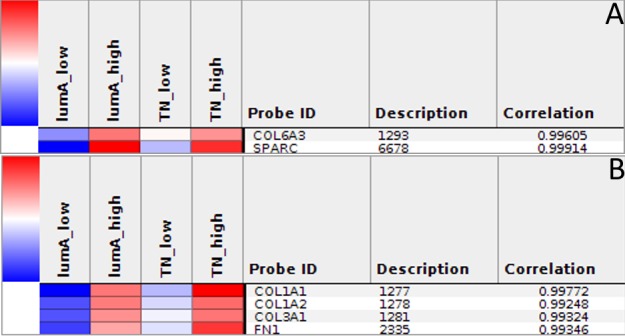
**A**. and **B**. Heatmap representing common profiles in *NOTCH3* lumA/TN unfavorable phenotypes.

The Express Cluster Analysis of Notch target genes identified unique expression profiles which differentiated lumA and TN BC. The clusters indicated differentially or equally-expressed genes among various good or bad prognosis phenotypes for lumA and TN subtypes. Heatmaps revealed changes in the expression of genes in lumA/TN *HES1*/*LFNG*/*PSEN1*/*ADAM10*/*NOTCH1*/*NOTCH3* good/poor prognosis groups (Supplementary File 8). Most notably, *COL18A1*, *DSP*, *ITGB1*, *MMP11*, *TAGLN* and *THBS2*, among others, were commonly upregulated in the lumA and TN *NOTCH1* poor prognosis phenotype (Figure 10), whereas *COL6A3*, *SPARC*, *COL1A1*, *COL1A2*, *COL3A1* and *FN1* were upregulated in the lumA/TN *NOTCH3* bad prognosis phenotype (Figure 11). Class comparisons, showing the gene expression profiles of *HES1* vs *LFNG* vs *PSEN1* and *NOTCH1* vs *NOTCH3* DFS prognosis groups are presented as Venn diagrams. *ADAM10* was excluded due to its outlier expression profile in lumA and TN.

**Figure 12:**
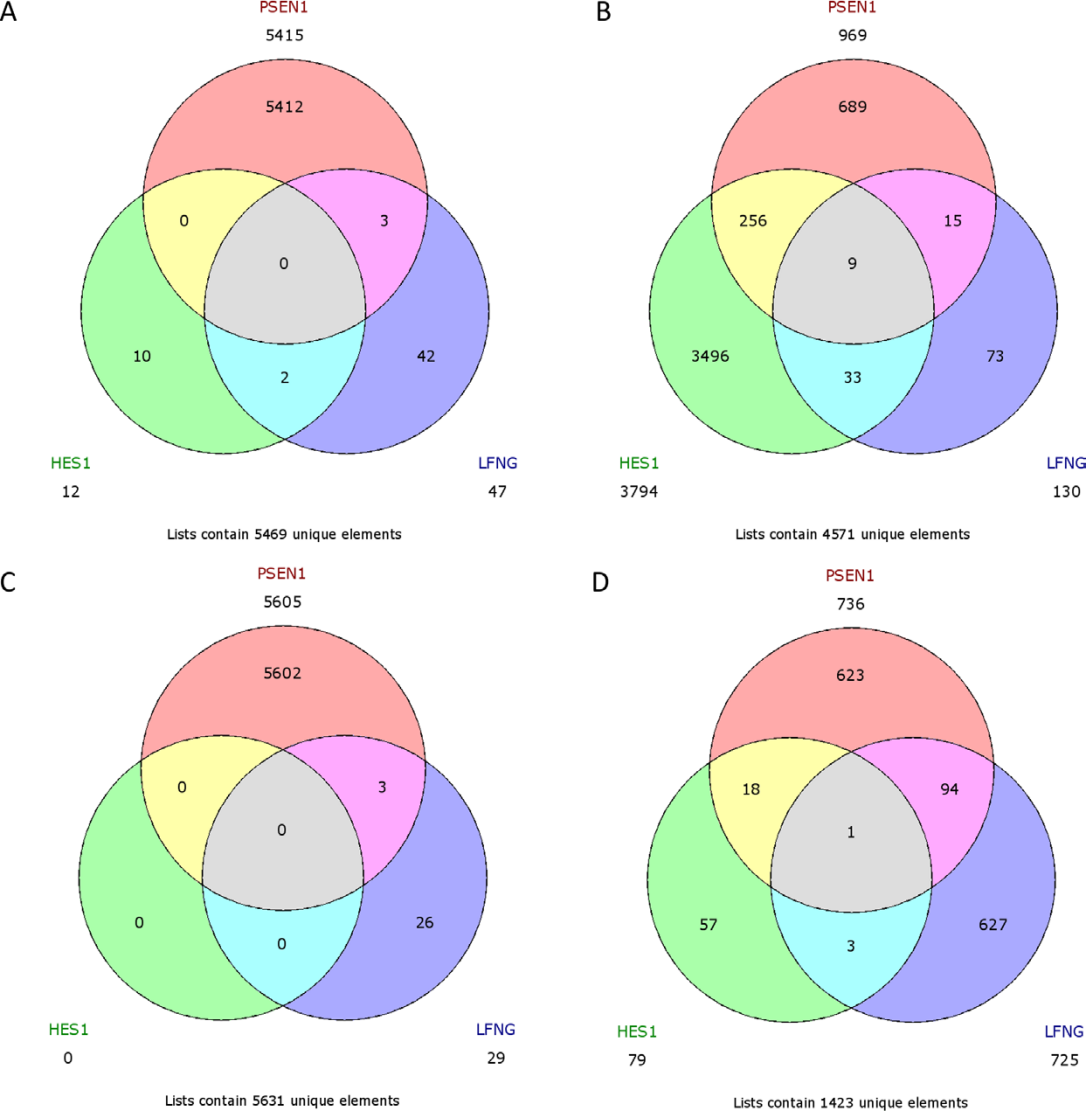
Venn diagrams representing class comparison of *HES1* vs *PSEN1* vs *LFNG* in favorable prognosis for **A**. upregulated genes, **B**. downregulated genes; and unfavorable prognosis for **C**. upregulated genes, **D**. downregulated genes.

**Figure 13:**
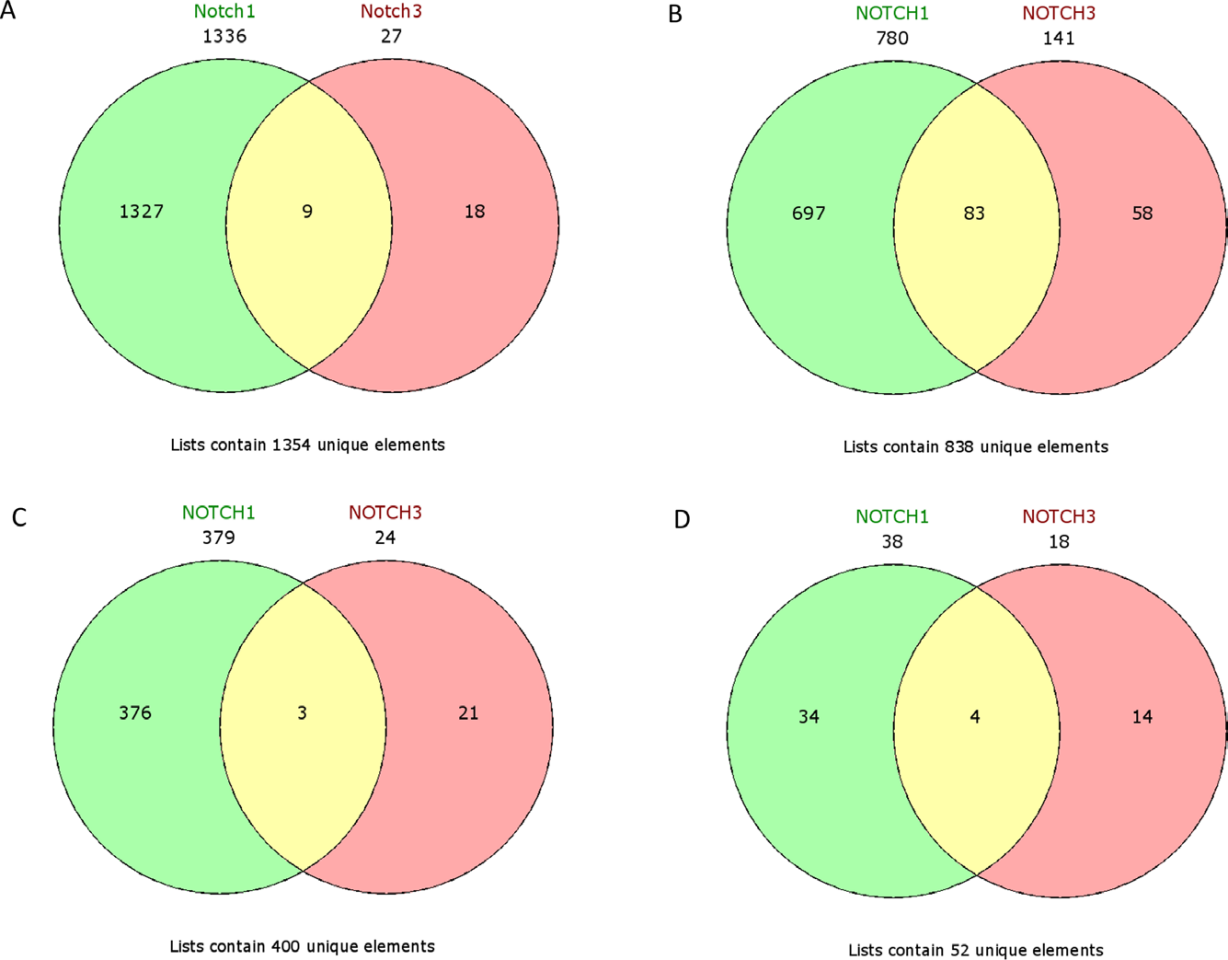
Venn diagrams representing class comparison of *NOTCH1* vs *NOTCH3* in favorable prognosis for **A**. upregulated genes, **B**. downregulated genes; and unfavorable prognosis for **C**. upregulated genes, **D**. downregulated genes.

No common upregulated genes were identified for the *HES1*/*LFNG*/*PSEN1* favorable prognosis, but nine common downregulated genes were found (*BCAP31*, *CALM1*, *FTL1*, *GNB2*, *NPC2*, *PRDX5*, *RAC1*, *SSR4*, *UQCRC1*) (Figure 12). Furthermore, only one downregulated common gene, *F11R*, was found in the *HES1*/*LFNG*/*PSEN1* unfavorable prognosis (Figure 12). The comparison of *NOTCH1*/*NOTCH3* revealed nine common upregulated genes (*AZIN1*, *CDC42*, *GOLGA4*, *H3F3A*, *KIF5B*, *PCMTD1*, *TM9SF3*, *TMED2*, *URB5*) and 83 downregulated common genes, including *COLA1A1*, *COL1A2*, *DST*, *FN1*, *RUNX1*, *TGFB1* and *SPARC* (Figure 13). Four downregulated genes (*BCAP31*, *HSPA5*, *PRDX1*, *SERP1*) and three upregulated genes (*MMP11*, *TAGLN*, *THB2*) were found for the *NOTCH1*/*NOTCH3* unfavorable prognosis (Figure 13).

Additionally, we performed cross - validation of our findings based on independent BC cohorts, however regarding many differences that occur within the data, the results cannot be compared. Uni - and multivariate Cox analyses showed that Notch signatures does not have independent prognostic value (see Supplementary Results).

## DISCUSSION

Our study evaluates the prognostic effect of the expression of Notch pathway members on DFS in lumA and TN BC. RNA-seq expression data obtained from tumor tissues was compared with the TCGA database, and the findings allowed patients to be assigned favorable / unfavorable prognosis based on aberrant Notch signaling. Although 19 genes involved in Notch pathway were initially examined, only 13 of them were found to be significantly associated with disease recurrence prognosis (Table 1).

*NOTCH1* is the best studied Notch receptor with regard to breast cancer. *NOTCH1* mutations have been reported in a high proportion of tumors, and are known to impair mammary stem cell self-renewal and promote cell transformation [23]. Furthermore, *NOTCH1* has been identified as a mediator of the *RAS* oncogenic pathway; this is often deregulated during the early stages of breast cancers and participates in the *JAG1*/*NOTCH1*/*CCND1* axis critical for maintaining proliferation of TN BC cells [24, 25]. Importantly, while high levels of NOTCH1 protein correlate with poorer patient prognosis [12], its mRNA level was not significantly associated with overall survival in BC [26].

Hu et al found *NOTCH3* to have transforming potential *in vivo*, as its activation led to tumor development [27]. In addition, *NOTCH3* activation has been detected in various breast cancer cell lines [28]. On the contrary, *NOTCH3* inhibition correlated with decreased osteoblast- and TGF-β-1- stimulated colony formation [29]. As expected, favorable DFS prognosis was observed to be associated with lowered expression of both *NOTCH1* and *NOTCH3* in lumA and TN BC (Figures 1 and 2).

To mediate Notch downstream signaling, the receptors must be processed by proteases. Recent studies indicate that ADAM10 is involved in the cleavage of a number of proteins such as the NOTCH receptor, its ligand DLL1 and other proteins influencing the metastatic potential of tumor cells through EMT (N-cadherin, E-cadherin, B-catenin). In particular, depending on its intracellular localization, B-catenin may play a dual role in epithelial cells: being a plasma membrane component and linking E-cadherin to the actin cytoskeleton, it is essential for adherens junction activity; on the other hand, it is also a major effector of the Wnt pathway and localizes to the nucleus after the loss or downregulation of E-cadherin expression, thus enhancing tumor aggressiveness and metastatic potential. Maretzky et al. have shown that ADAM10 modulates B-catenin singling via regulation of cell surface exposition of E-cadherin, therefore affecting the expression of B-catenin downstream targets [30–32]. *ADAM10* is also involved in EGFR and ERBB2 receptor shedding, thus demonstrating its critical role in breast cancer [33]. To date *ADAM10* overexpression has been identified in several malignancies [34–36]. In a study of its clinical potential in breast tumors, Feldinger et al. found high *ADAM10* expression to be associated with poorer trastuzumab response and worse relapse-free survival in HER2+ BC [37]. Nevertheless, it is not clear whether *ADAM10* expression has a prognostic role in lumA and TN BC. Our results indicate that lowered expression of *ADAM10* is favorable for DFS in lumA whereas high expression is favorable in TN BC (Figures 1 and 2). Importantly, the finding that the *CTNNB1* oncogenic signature gene set is upregulated in lumA *ADAM10* unfavorable prognosis groups indirectly indicates the presence of cross-talk between *ADAM10* and B-catenin (Table 4).

LFNG is a β3N-acetylglucosaminyl-tranferase, which regulates ligand-mediated activation of the Notch pathway: it enhances Notch activation through Delta-like ligands (DLL1, DLL4) and inhibits its activity through Serrate/Jagged ligands (JAG1, JAG2) [38]. Raouf et al. suggest that the expression of *DLL1* in myoepithelial cells activates Notch in the *LFNG*-expressing mammary stem cells (MaSCs) and bipotent progenitor cells present in the human breast. High *JAG1* expression has been found in the epithelial compartment; the lowered *LFNG* level thus increases Jagged-activated Notch signaling and induces the proliferation of luminal progenitors, which has been associated with TN tumors [39, 40].

*HES1* is transcription repressor and downstream effector of Notch signaling. *HES1* has been proposed as an indicator of Notch signaling activity in many cancers [41]. However, its molecular activity depends on the context [42]. It has been shown that in ER+ BC, estrogen promotes the activation of Notch signaling through *JAG1* and represses *HES1* expression, leading to increased cell proliferation [43]. Moreover, some studies have indicated that *HES1* is able to inhibit Notch signaling via repression of its ligands (*JAG1*, *DLL1*), implying possible negative feedback regulation of the Notch pathway [44, 45].

During Notch activation, several proteolytic processing steps occur. Presenilin 1 (PSEN1) is member of the γ-secretase complex involved in the proteolysis of the Notch intermediate peptide, termed Notch extracellular truncation (NEXT) [9]. The significance of PSEN1 in pathology has been widely presented in Alzheimer's disease and other neurodegenerative disorders, as it generates amyloid β [46]. However, its role and prognostic value in breast cancer remains unclear. Nevertheless, Rizzo et al. report that estrogen inhibits Notch signaling through inhibition of Notch receptor cleavage by the γ-secretase complex. In addition, loss of estrogen caused by estrogen deprivation or antiestrogen treatment in neoplastic cells results in enhanced proliferation, survival and invasion as an effect of *NOTCH1* reactivation; in contrast to ER- cells, normal ER+ breast cells are non-proliferative [47].

Until now, the prognostic values of *LFNG*, *HES1* and *PSEN1*, and the relationship between their mRNA level with BC DFS have not been evaluated; however, the present study has two key novel findings: firstly, that an elevated level of *LFNG*, *HES1* and *PSEN1* has a favorable effect in lumA BC, as predicted, while lowered *LFNG*, *PSEN1* and *HES1* expression correlated with better prognosis in TN BC (Figure 2). Additionally, Notch ligand expression (*JAG1*, *JAG2*, *DLL4*) was not found to have any significant effect on DFS in TN BC. Together with lowered *NOTCH1* and *NOTCH3*, those results indicate inferior activation of the Notch pathway in the favorable prognosis group in TN BC. Moreover, although both BC subtypes are classified as HER2-, they differ in estrogen/progesterone receptor status, tumor biology and clinical course of disease. Such differences in the favorable expression of *HES1*, *LFNG* and *PSEN1* in lumA and TN may be attributable to variation in the compensative influence of remaining Notch members, or the activation or inhibition of additional pathways.

Sørlie et al. outlined five intrinsic subtypes of BC that differ in clinical outcomes and tumor biology [4]. In particular, lumA cancer cells mimic the luminal epithelial components of the breast (ER+ PR+ HER2-) and are characterized by favorable overall prognosis; however, the risk of recurrence is correlated with metastasis to lymph nodes by the time of diagnosis. In contrast, TN cancer cells mimic basal epithelial cells and normal breast myoepithelium (ER- PR- HER2-), and patients face a poor prognosis [48].

In our study we have appliedGSEA to compare DFS groups in patients with lumA and TN BC. Various sets of genes associated with TF binding motifs were found to be upregulated according to disease recurrence prognosis (Table 2).

The E2F family members are important regulators of the cell cycle [49]. They have been demonstrated to be involved in the regulation of apoptosis and proliferation in human cancers [50]. Hollern et al. reported that loss of E2Fs enhanced ductal transformation and tumor onset *in vivo*, and that E2Fs mediate the expression of genes critical to angiogenesis, tissue and cell remodeling, and interactions between tumor cells and vascular endothelium to facilitate lung metastasis [51]. Our results indicate that E2F target gene sets were upregulated in the *HES1* and *PSEN1* lumA bad prognosis and TN good prognosis groups, as well as in the *NOTCH1* bad prognosis group in both subtypes (Figures 3-7). As activation of the Notch pathway via *NOTCH1* is known to be unfavorable in both subtypes, upregulated targets of E2Fs may be associated with an enhanced recurrence rate. In contrast, other genes in the same gene set were upregulated in lumA bad and TN good prognosis groups. These results demonstrate the biological differences underlying lumA and TN subtypes affecting cell cycle regulation, proliferation and apoptosis through E2Fs; however, these associations should be further investigated.

*E2F1* is a transcription activator belonging to the E2F family. The *E2F1* and *SP1* gene target sets were found to be upregulated in the *ADAM10*, *NOTCH1* and *NOTCH3* lumA unfavorable phenotype (Figures 5 and 6); however, *SP1* transcription factor is known to be a global regulator of cellular differentiation. The associations of *E2F1*, *SP1* and estrogen receptor in breast cancer have been described previously [52, 53]. It was found that higher expression of *E2F1* in ER+ BC (i.e. lumA) enhances tamoxifen resistance through SP1-ERα interactions promoting recruitment to the proximal promoter of *E2F1 in vitro* [53]. In contrast, overexpression of *E2F1* and its target genes was found to positively influence E2F1-mediated cell death in ER- breast cancer cells *in vitro* [52]. Our results indicate that the enhanced expression of *E2F1* and *SP1* target genes plays a role in the unfavorable lumA phenotype. Furthermore, we observed upregulation of different genes within the same *SP1* gene set in *ADAM10* lumA good prognosis group, but at a considerably lower level (Figure 6), hence revealing significant differences in cellular biological mechanisms between favorable and unfavorable phenotypes.

*GATA3* is a transcription factor belonging to the GATA family, which is essential for cell-fate specification, i.e. luminal epithelial cell differentiation [54]. Moreover, *GATA3* expression is favorable during carcinogenesis as it impedes the EMT and inhibits the metastasis of cancer cells [55]. Conversely, a lack of GATA3 leads to drug-resistance and a mesenchymal-like phenotype [56]. Our results show upregulation of *GATA3* target genes in *ADAM10*, *NOTCH1* and *NOTCH3* lumA bad prognosis groups (Figure 7), but no significant upregulation in the TN subtype. *GATA3* expression has been shown as impeding EMT; however, its upregulated targets may be somehow associated with a worse recurrence prognosis. Most importantly, our GSEA results reveal upregulation in gene sets associated more closely with cells undergoing EMT or with an executed mesenchymal phenotype among bad prognosis groups (Table 3). Our findings demonstrate the presence of unfavorable events typically associated with the transition between epithelial to mesenchymal phenotypes in bad prognosis groups of *NOTCH1*, *NOTCH3* and *ADAM10* in both subtypes.

Therefore, we assumed that worse prognosis stems from the potential of cells to switch to a less favorable mesenchymal phenotype; our findings revealed an upregulation of gene sets regarding canonical pathways, biological processes and molecular functions indicating EMT. Among the gene sets upregulated in the *NOTCH1*, *NOTCH3* and *ADAM10* unfavorable prognosis groups, a number of molecular markers of the mesenchymal phenotype were found to be not upregulated in good prognosis groups: *VIM*, *MMP2*, *CDH2*, *ITGA5*, *FN1* and *SPARC* (Table 3). CGPs demonstrated differences in resistance or sensitivity to various treatment regimens according to prognosis group. In accordance with previous results, a common treatment response profile was found for the *ADAM10*, *NOTCH1* and *NOTCH3* unfavorable prognosis groups (Table 4). In addition, our initial assumptions were confirmed by the “metastasis through EMT” gene set being upregulated.

The study also evaluated the influence of the *ADAM10*, *NOTCH1*, *NOTCH3*, *HES1*, *LFNG* and *PSEN1* genes on breast cancer recurrence. Cluster analysis was used to evaluate the common and unique expression profiles of genes transcriptionally activated by Notch TFs, such as *HES1* and *HEY1* (Supplementary File 8). Specifically, integrin, metalloprotease, collagen and desmoplakin genes involved in EMT were found to be activated; their expression indicated a mesenchymal phenotype in bad prognosis groups, that transition was in progress, or the presence of single changes associated with primary potential to undergo EMT (Figures 8 and 9, Table 5).

A class comparison was performed to compare genes associated with the studied phenotypes, with the results presented as Venn diagrams. *ADAM10* group was excluded due to its outlier expression profile. The *HES1*/*LFNG*/*PSEN1* favorable prognosis groups possessed no common upregulated genes but nine common downregulated genes (Figure 12), while the unfavorable prognosis groups only had one downregulated gene in common (*F11R*): a clear biological difference between lumA and TN tumors, as well as between Notch members. The *NOTCH1*/*NOTCH3* unfavorable groups were found to have three common upregulated genes (*MMP11*, *TAGLN*, *THB2*) (Figure 13).

In summary, although the biology of BC has been well established, there is a lack of knowledge concerning the regulation of specific signaling pathways, as well as useful prognostic biomarkers, especially for DFS prognosis. The mechanisms of recurrence and roles of Notch in tumourigenesis of the breast are still unclear. Our findings indicate that the expression profiles of Notch pathway members can be used to differentiate the DFS in lumA and TN BC subtypes, and so may serve as novel prognostic biomarkers. Moreover, the study highlights significant new differences in the biology of the two tumors, and indicates that differences in the signals activating the Notch pathway result in the occurrence of common aberrant mechanisms, such as triggering EMT. It seems that aberrant expression and regulation of Notch receptors has the most significant influence on the course of disease. Notably, our results indicate that while there are subgroups of patients who will probably never experience disease relapse, other subgroups exist within the lumA subtype which have a higher risk of recurrence due to potential transition into mesenchymal cell type. Finally, it was found that *MMP11*, *TAGLN* and *THB2*, three genes involved in acquiring mesenchymal phenotype and which are regulated by the Notch pathway, can be used as potential therapeutic targets.

## MATERIALS AND METHODS

The RNA-seq profiling (level 3 RNASeqV2, RSEM normalized) and clinical data of 1098 BC patients was obtained from The Cancer Genome Atlas (TCGA) data portal (http://cancergenome.nih.gov/, data status of Jan 28, 2016). The methods of biospecimen procurement, RNA isolation and RNA sequencing were previously described by The Cancer Genome Atlas Research Network [57].

The TCGA RNA-seq data was cross-referenced with the clinical information of the patients. Patients with missing clinical/expression values were excluded from further analyses. A total of 1081 samples were included in the study. The clinical characteristics of cohort patients are presented in Table 6.

**Table 6:** Clinical characteristics of lumA and TN BC cohort patients

Characteristic	lumA		TN	
	Total	%	Total	%
Age at diagnosis				
median age (range)	58 (28 – 90)		53.5 (29 – 90)	
Race				
White	270	73.6	68	59.6
Asian	21	5.7	8	7
Black or African American	29	7.9	31	27.2
NA’s	47	12.8	7	6.1
Menopause status ^1^				
premenopausal	88	24	30	26.3
perimenopausal	16	4.4	5	4.4
postmenopausal	235	64	69	60.5
indeterminate	1	0.3	2	1.8
NA’s	27	7.4	8	7
Stage				
I	72	19.6	20	17.5
II	202	55	70	61.4
III	84	22.9	19	16.7
IV	3	0.8	2	1.8
x	5	1.4	-	-
NA’s	1	0.3	3	2.6
Histology				
infiltrating ductal carcinoma	243	66.2	97	85.1
infiltrating lobular carcinoma	88	24	3	2.6
metaplastic carcinoma	1	0.3	5	4.4
mucinous carcinoma	12	3.3	-	-
medullary carcinoma	-	-	2	1.8
mixed histology	9	2.5	1	0.9
other	14	3.8	5	4.4
NA’s	-	-	1	0.9
Therapy type				
chemotherapy	149	40.6	81	71.1
hormone therapy	119	32.4	-	-
immunotherapy	2	0.5	-	-
other	1	0.3	2	1.8
NA’s	96	26.2	31	27.2
Primary lymph node presentation				
positive	222	60.5	73	64
negative	14	3.8	3	2.6
NA’s	131	35.7	38	33.3

The clinical characteristics shown here are in whole based upon data generated by the TCGA Research Network: http://cancergenome.nih.gov/

^1^ “Premenopausal” status defined as <6 months since last menstrual period (LMP) and no prior bilateral ovariectomy and not on estrogen replacement; “perimenopausal” status defined as 6-12 months since LMP; “postmenopausal” status defined as prior bilateral ovariectomy or >12 months since LMP with not prior hysterectomy; “indeterminate” status defined as neither pre- or postmenopausal.

To identify lumA and TN BC subtypes, the data was subsampled according to the following clinical parameters: “patient.breast_carcinoma_estrogen_receptor_status” for ER distribution, “patient.breast_carcinoma_progesterone_receptor_status” for PR distribution and “patient.lab_proc_her2_neu_immunohistochemistry_receptor_status” for HER2/neu distribution. Finally, patients with ER+PR+HER2- characteristics were classified as the lumA subgroup (367 patients) and ER-PR-HER2- (114 patients) as the TN subgroup.

Among all breast cancer patients, groups of lumA and TN BC data were identified to determine whether differential expression of 19 Notch signaling pathway members is associated with cancer recurrence. The analyzed genes and their functions in the Notch pathway are listed in Table 7. The analysis was based on optimal cutoff point determination, which enabled patients to be categorized according to favorable or unfavorable prognosis based on the expression of Notch members. The analysis was performed separately for each cancer subtype using the Cutoff Finder web application (http://molpath.charite.de/cutoff/). The clinical characteristics defining DFS were “patient.days_to_last_followup” for survival time and “patient.follow_ups.follow_up.person_neoplasm_cancer_status” for outcome and event.

**Table 7:** Notch pathway members and their functions used in the study

Gene		
Symbol	Name	Function
*ADAM10*	Disintegrin and metalloproteinase domain-containing protein 10	Notch activatormetalloproteinase
*ADAM17*	Disintegrin and metalloproteinase domain-containing protein 17	
*APH1B*	γ-secretase subunit APH-1B	enzyme modulator
*DLK1*	Protein delta homolog 1	non-canonical Notch ligand
*DLL4*	Delta-like protein 4	canonical Notch ligand
*HES1*	Transcription factor HES-1	transcription factor
*HES4*	Transcription factor HES-4	
*HES5*	Transcription factor HES-5	
*HEY1*	Hairy/enhancer-of-split related with YRPW motif protein 1	
*JAG1*	Protein jagged-1	mediator of Notch signalling
*JAG2*	Protein jagged-2	
*LFNG*	β-1,3-N-acetylglucosaminyltransferase lunatic fringe	Notch regulator
*NOTCH1*	Neurogenic locus notch homolog protein 1	receptor
*NOTCH2*	Neurogenic locus notch homolog protein 2	
*NOTCH3*	Neurogenic locus notch homolog protein 3	
*NOTCH4*	Neurogenic locus notch homolog protein 4	
*NUMB*	Protein numb homolog	Notch antagonist
*PSEN1*	Presenilin-1	γ-secretase complex member
*PSEN2*		Presenilin-2

The descriptions of particular genes have been obtained from NCBI Gene Database: https://www.ncbi.nlm.nih.gov/gene/

The significance of correlation with survival variable was chosen as the method for cutoff point optimization, briefly defined as the point with the most significant split. Additionally, hazard ratios (HRs) including 95% confidence intervals (CI) were calculated [58]. Differences in DFS between the Favorable and unfavorable groups, defined by the computed cutoff point for Notch member expression, were depicted using Kaplan-Meier curves with calculated p-values (log-rank test, p<0.05).

GSEA was performed to determine the biological significance in terms of KEGG canonical pathways, CGP, TF binding motifs and gene ontology (GO): biological processes (BP), cellular components (CC), and molecular functions (MF) [59]. Enrichment analysis was performed for 20502 genes. Phenotype labels, defined as good or bad prognosis according to the computed cutoff point for each Notch pathway member, were determined for both lumA and TN BC. Additionally, to elicit the most relevant associations of differential Notch signaling, groups of patients with the extreme values of particular Notch member expression were chosen (first and fourth quartile regarding the expression level). Enrichment was subjected to GSEA by applying tTest with a weighted scoring scheme and permutation type regarding phenotype, using the significance threshold of FDR<0.25.

ExpressCluster software (http://cbdm.hms.harvard.edu/) was used to find common and unique expression profiles of genes activated by Notch transcription factors (HES, HEY families). A total of 9346 *HES1* and *HEY1* targets were extracted from MSigDB on the basis of presence of binding motifs for both TFs. Clustering was performed by applying the K-means algorithm, mean centered signal transformation and Euclidean distance metric. Profiles indicating contrasts between lumA and TN BC or genes associated with favorable/unfavorable prognosis were considered as significant.

Further associations between common and contrasting genes were visualized using the VennDiagram Generator web application (http://www.bioinformatics.lu/venn.php).

Additionally, we performed cross - validation of our findings based on independent BC cohorts obtained from USCS Xena as well as uni - and multivariate Cox analyses to assess if any of clinical characteristics including Notch signaling may have independent prognostic value. Further details may be found in Supplementary Materials.

## SUPPLEMENTARY MATERIALS FIGURES AND TABLES












