# Supplementary material for: Common profiles of Notch signaling differentiate disease-free survival in luminal type A and triple negative breast cancer
Source: Oncotarget. 2016 Nov 19;8(4):6013–32. doi: 10.18632/oncotarget.13451 (PMC5351609; doi:10.18632/oncotarget.13451)
Supplement: Supplementary file 1 [file oncotarget-08-6013-s001.pdf]

Common profiles of Notch signaling differentiate disease-free survival in luminal type A and triple negative breast cancer

SUPPLEMENTARY DATA

SUPPLEMENTARY RESULTS

Cross - validation of primary findings

Analysis of Caldas cohort confirmed our findings that lower expression of *NOTCH1* and *NOTCH3* was tendentiously lower favorable for disease free survival, albeit the results were not statistically significant (Supplementary Figures 1 and 2). In contrast, Chin cohort analysis showed that higher expression of *NOTCH1* and *NOTCH3* were favorable for disease – free survival, albeit only *NOTCH3* showed significant correlation (Supplementary Figures 3 and 4). The latter stands in opposition to our primary findings, although there are several critical differences between TCGA and Chin cohorts that may affect the final data. Besides the basic differences between the cohorts (including ethnicity, race etc), the most important is the use of different platforms such as sequencing vs microarrays. According to Tian et al. Paper the microarray data are much more biased with extensive mathematical corrections than RNAseq, therefore there is only 67 – 68% average reproducibility between the technologies (*Tian F, Wang Y, Seiler M, Hu Z. Functional characterization of breast cancer using pathway profiles. BMC Med Genomics. 2014 Jul 21; 7:45*). In addition, RNAseq presents the absolute measurement of expression level, which cannot be obtained using microarray technology, therefore cutoff points computed by Cutoff Finder for validation purposes are not comparable due to negative values (Supplementary Figures 1–4).

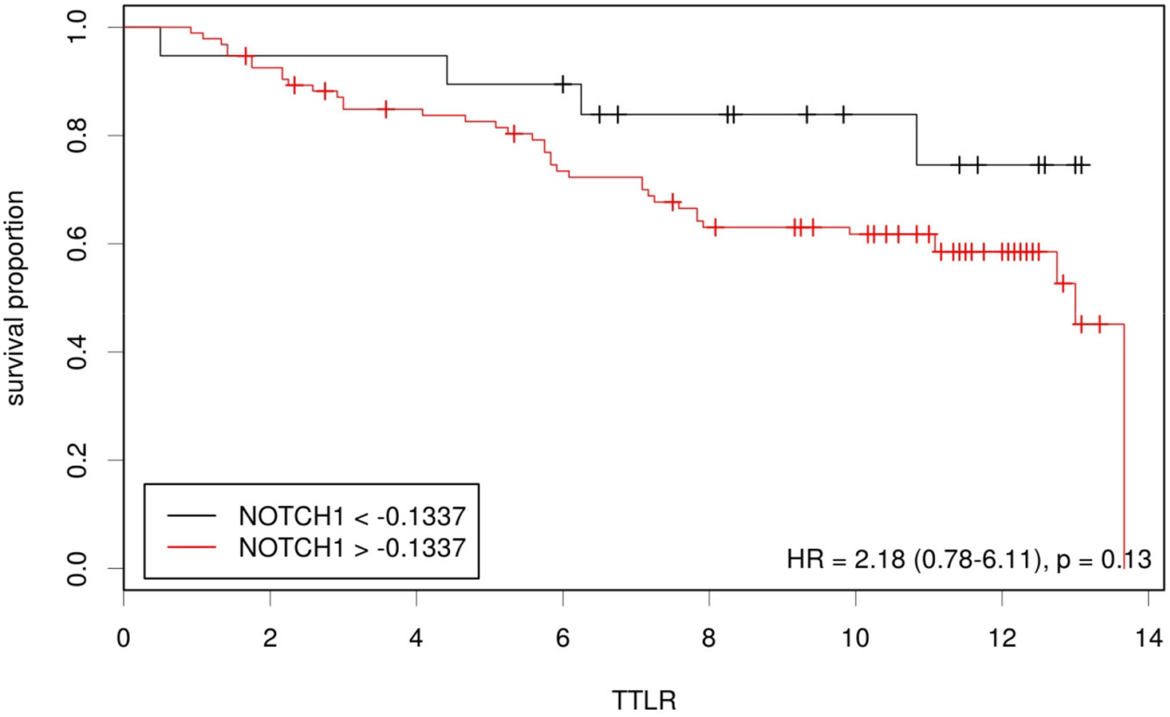

Supplementary Figure 1: Kaplan - Meier plot for *NOTCH1* based on Caldas cohort.

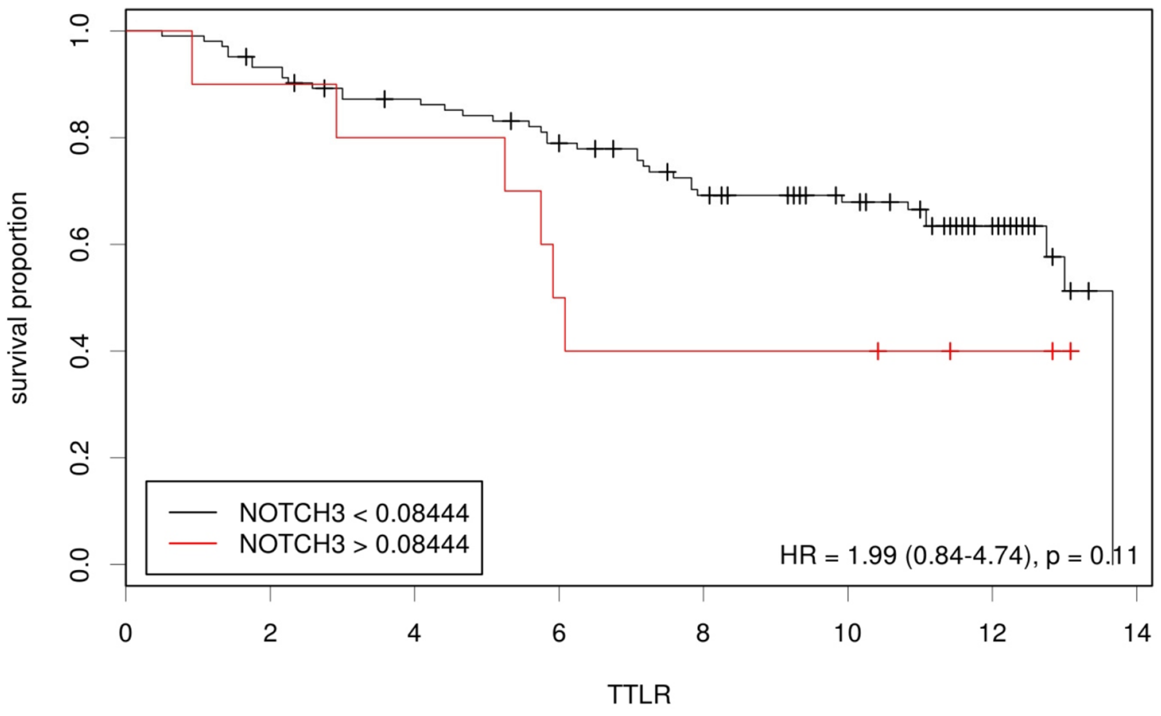

Supplementary Figure 2: Kaplan - Meier plot for *NOTCH3* based on Caldas cohort.

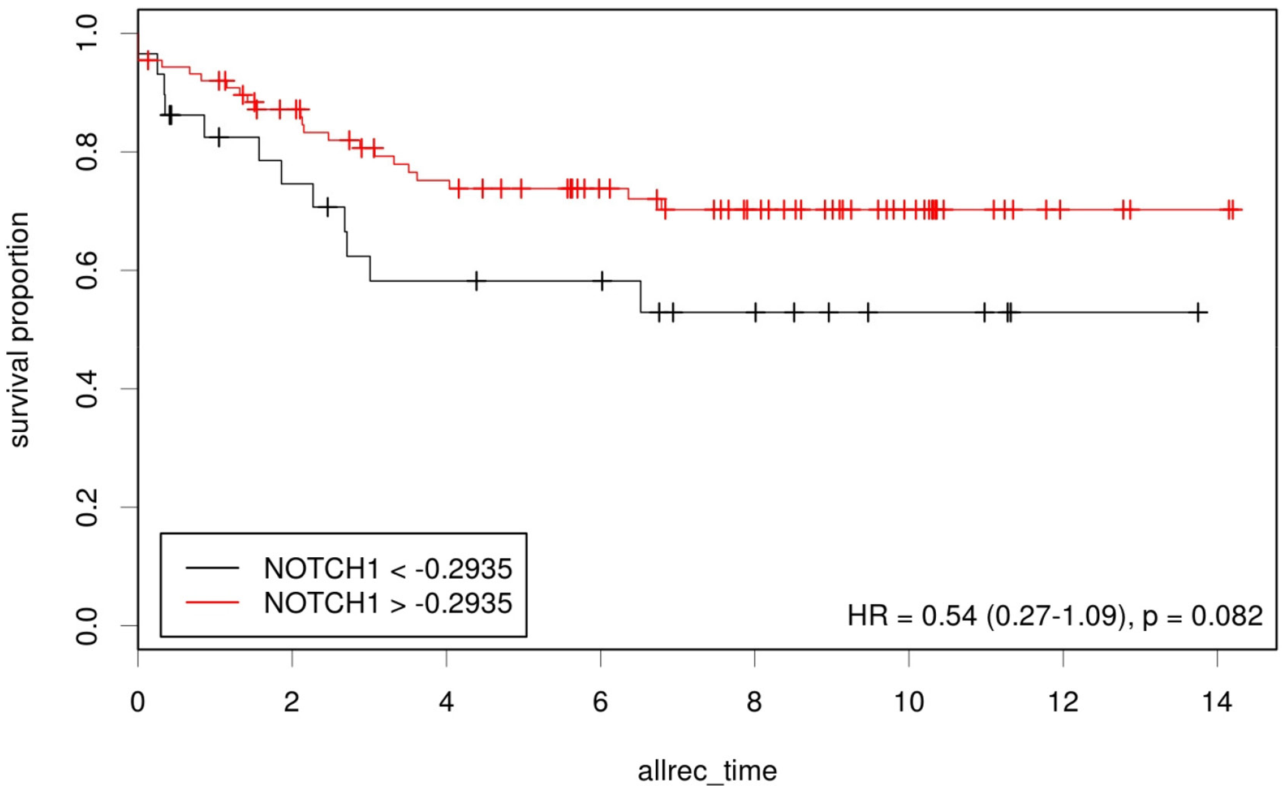

Supplementary Figure 3: Kaplan - Meier plot for *NOTCH1* based on Chin cohort.

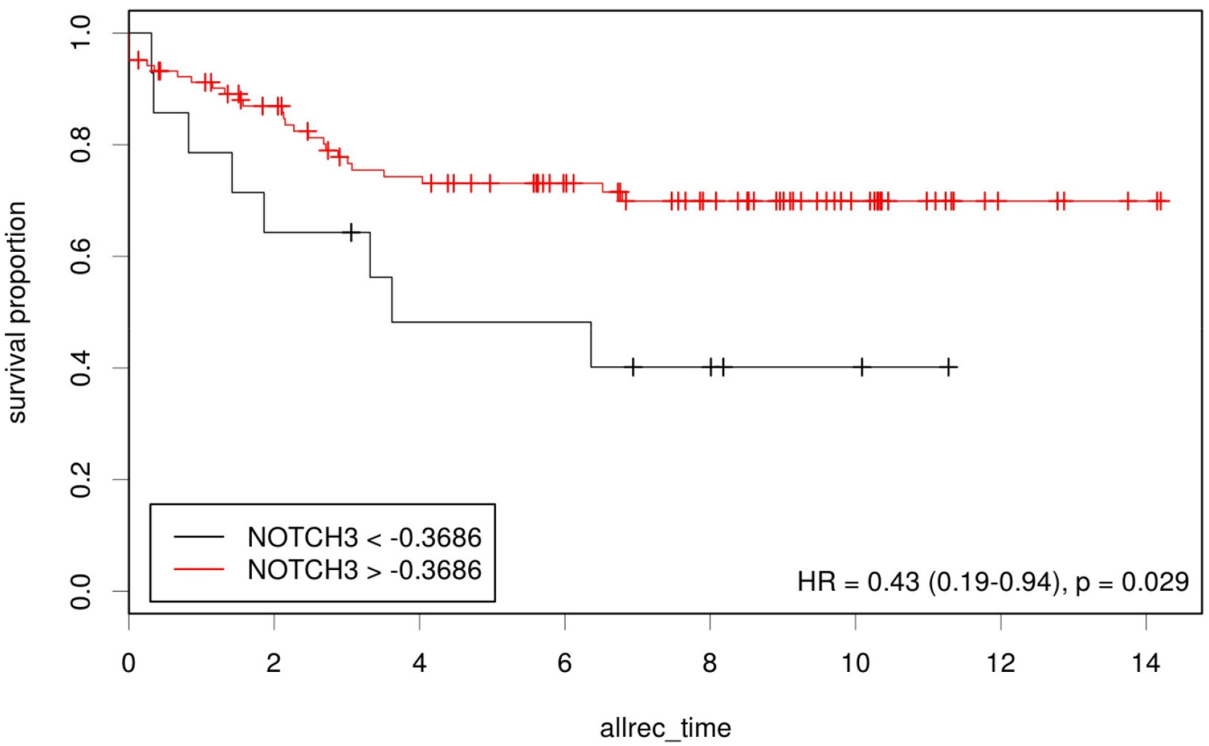

Supplementary Figure 4: Kaplan - Meier plot for *NOTCH3* based on Chin cohort.

Uni- and multivariate Cox proportional hazards model analyses

We performed both uni - and multivariate Cox analyses to assess if any of the patients clinical characteristics including Notch members may be considered as independent prognostic value separately for lumA and TN BC. We found that none of the studied factors nor identified Notch signature do not have independent prognostic value (Supplementary Tables 1 and 2).

Supplementary Table 1: Uni- and multivariate Cox analyses for lumA BC

See Supplementary Table 1

Supplementary Table 2: Uni- and multivariate Cox analyses for TN BC

See Supplementary Table 2

SUPPLEMENTARY METHODS

We performed the attempt to cross – validate our findings (DFS analysis) using independent breast cancer cohorts. We employed studies of Caldas (2007) and Chin (2006) obtained from UCSC Xena (http://xena.ucsc.edu). In fact, those data missed relevant information including the distribution of hormone receptors. Therefore, we were not able to classify patients to luminal type A or triple negative breast cancers. Regarding the above we performed the analysis for only two genes – *NOTCH1* and *NOTCH3*. Due to deficiency in necessary parameters we were not able to split patients into breast cancer subgroups, therefore we focused on those two genes, which were significant regardless to cancer subtype for general comparison of our findings with independent study. Among chosen cohorts we used the following data : *NOTCH1* and *NOTCH3* expression, "recurrence" to determine the relapse of the disease and "time to local recurrence" to define the time (Caldas, 2007), and "All Rec" to determine the relapse of the disease and "All Rec Time" to define the time (Chin, 2006). Subsequently, we performed the DFS analysis separately for each cancer subtype using Cutoff Finder. Clinical characteristics defining DFS regarding *NOTCH1* and *NOTCH3* genes were as follows: "time to local recurrence" for survival time and "recurrence" for outcome and event in Caldas cohort as well as "All Rec Time" for survival time and "All Rec" for outcome and event in Chin cohort. We chose significance of correlation with survival variable as a method for cutoff point optimization. Differencesin DFS between "favourable" and "unfavourable" groups (defined by computed cutoff point for Notch members expression) have been presented in form of Kaplan-Meier plots with p-values calculated (log-rank test, $p<0.05$).

In addition, we performed uni- and multivariate Cox proportional hazards model analyses to assess whether any of the patients clinical characteristics including Notch members may be considered as independent prognostic factor. For this purpose we employed survival R package including coxph() function.

Supplementary File 1: TF binding motifs in lumA unfavourable prognosis groups

See Supplementary File 1

Supplementary File 2: GO BP, CC, MF and KEGG canonical pathways in lumA unfavourable prognosis groups

See Supplementary File 2

Supplementary File 3: CGPs in lumA unfavourable prognosis groups

See Supplementary File 3

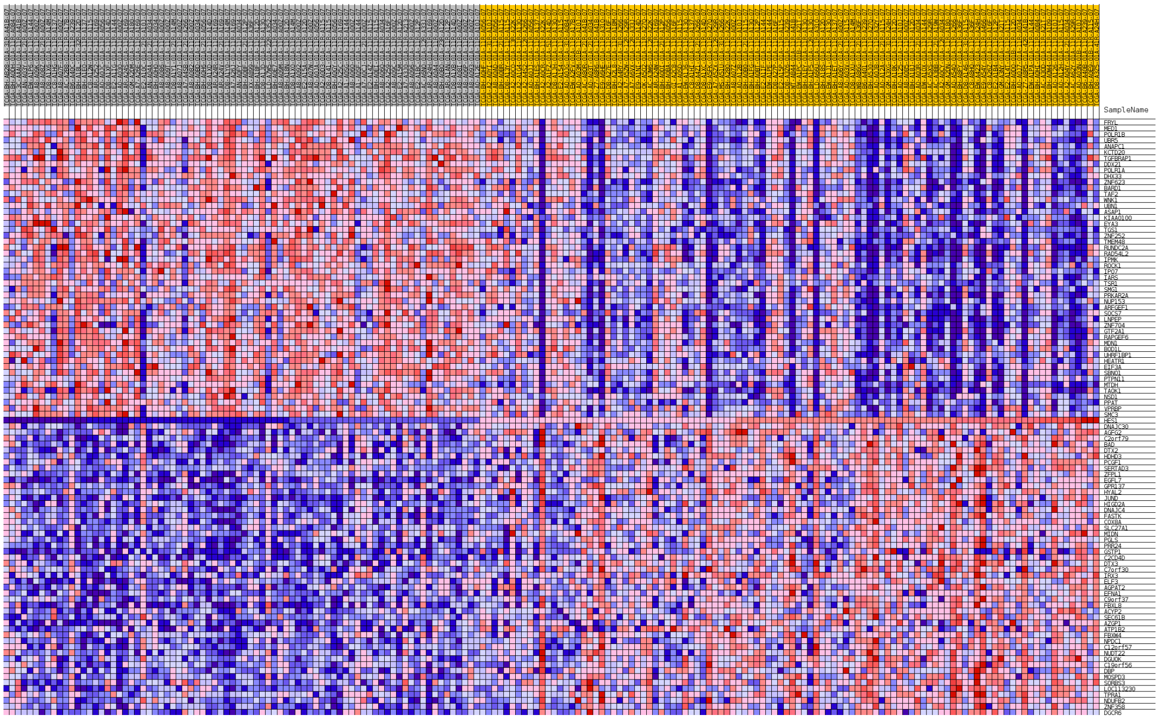

Supplementary File 4: Heatmap of 50 mostly differentiating marker genes for HES1 lumA phenotypes.

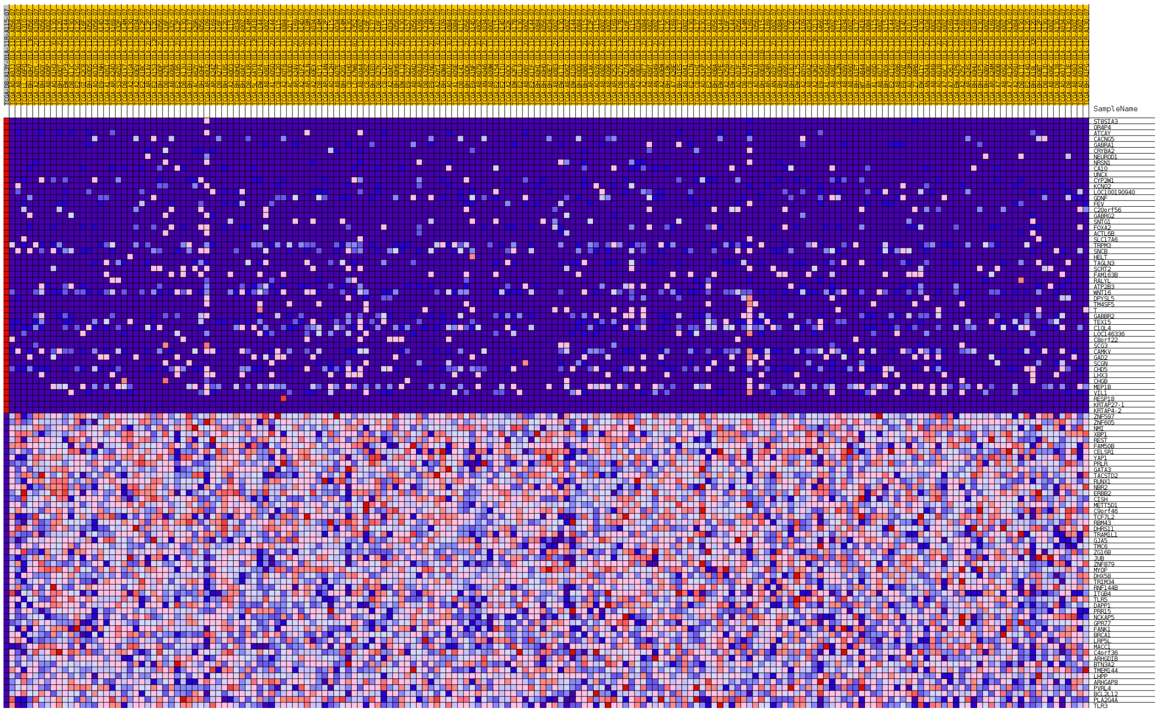

Supplementary File 5: Heatmap of 50 mostly differentiating marker genes for PSEN1 lumA phenotypes.

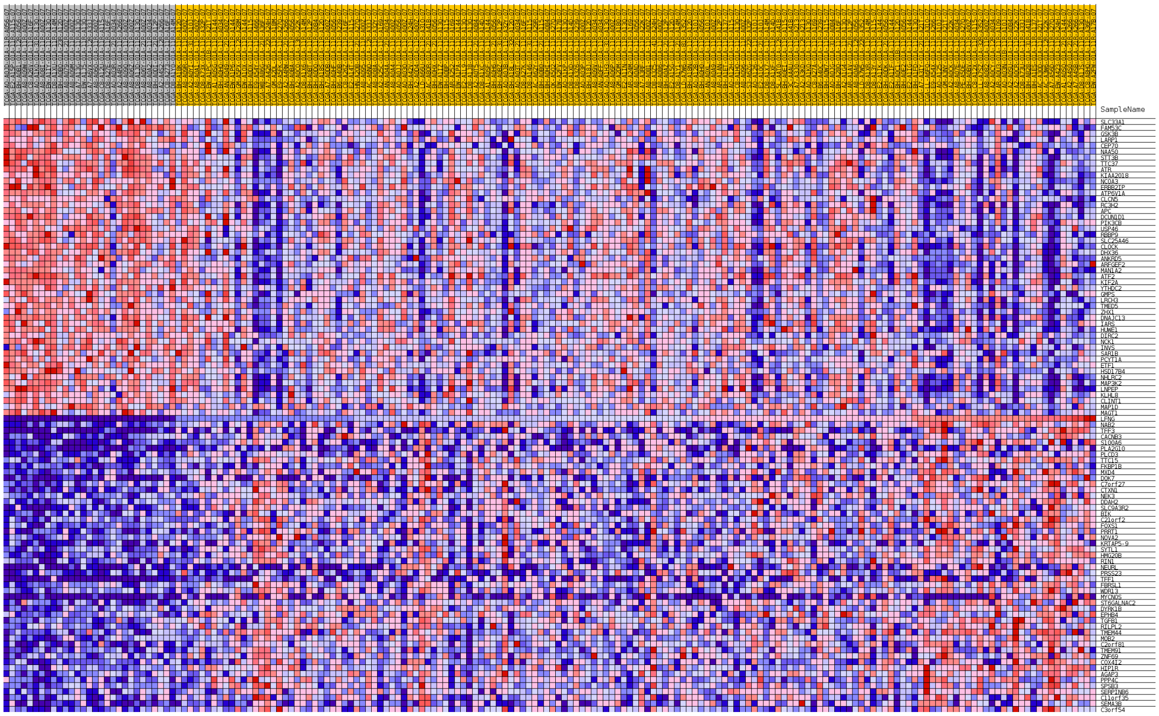

Supplementary File 6: Heatmap of 50 mostly differentiating marker genes for LFNG lumA phenotypes.

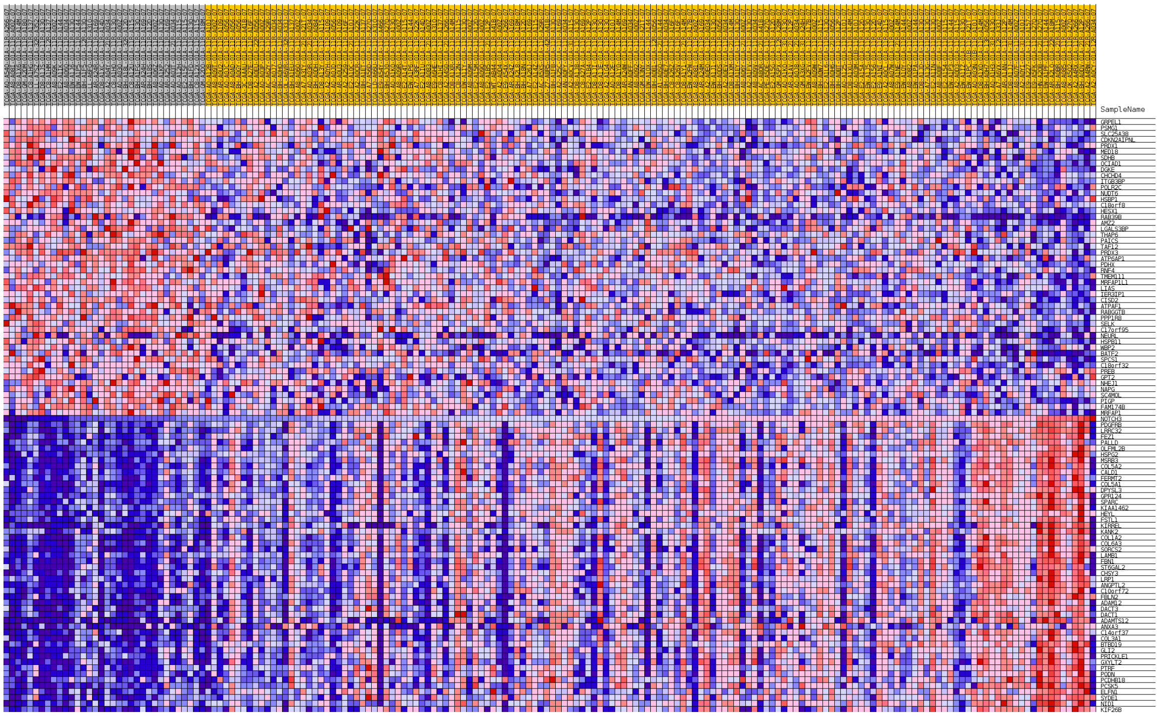

Supplementary File 7: Heatmap of 50 mostly differentiating marker genes for NOTCH3 lumA phenotypes.

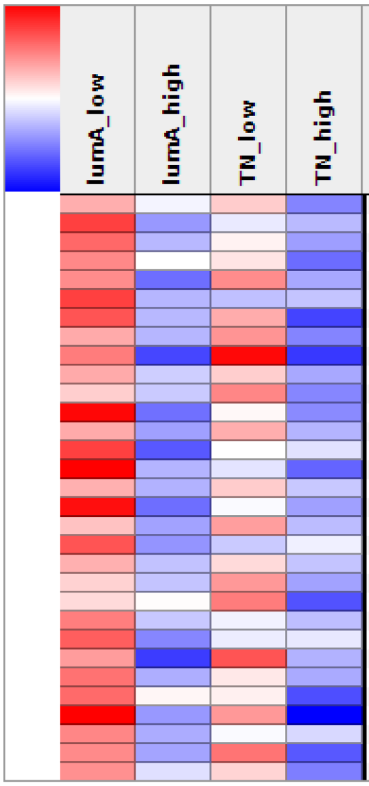

Probe ID	Description	Correlation
ARF4	378	0.88831
ARL6IP1	23204	0.89636
ATP5H	10476	0.97316
ATP6V0E1	8992	0.87539
BCAP31	10134	0.92469
CAPNS1	826	0.79388
EIF2AK1	27102	0.97294
EIF2S3	1968	0.92300
EPCAM	4072	0.87110
GFPT1	2673	0.98672
GGCT	79017	0.81947
H3F3A	3020	0.95073
HM13	81502	0.94869
HNRNPC	3183	0.84942
HSPA9	3313	0.91023
KDELR1	10945	0.97012
NACA	4666	0.92873
NDUFA4	4697	0.87870
PRDX2	7001	0.74486
PSMA6	5687	0.99478
RAB7A	7879	0.84198
RAC1	5879	0.68460
RBBP7	5931	0.91713
RPL14	9045	0.82756
RPL24	6152	0.82419
RTN3	10313	0.98416
SEPHS2	22928	0.84210
SERP1	27230	0.97835
ST13	6767	0.90375
TMED2	10959	0.92850
VAMP8	8673	0.94863

NOTCH1 favorable (lumA low; TN low)

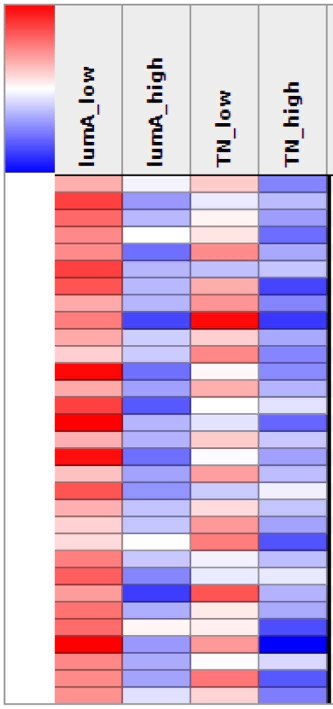

Probe ID	Description	Correlation
ARF4	378	0.88831
ARL6IP1	23204	0.89636
ATP5H	10476	0.97316
ATP6V0E1	8992	0.87539
BCAP31	10134	0.92469
CAPNS1	826	0.79388
EIF2AK1	27102	0.97294
EIF2S3	1968	0.92300
EPCAM	4072	0.87110
GFPT1	2673	0.98672
GGCT	79017	0.81947
H3F3A	3020	0.95073
HM13	81502	0.94869
HNRNPC	3183	0.84942
HSPA9	3313	0.91023
KDELR1	10945	0.97012
NACA	4666	0.92873
NDUFA4	4697	0.87870
PRDX2	7001	0.74486
PSMA6	5687	0.99478
RAB7A	7879	0.84198
RAC1	5879	0.68460
RBBP7	5931	0.91713
RPL14	9045	0.82756
RPL24	6152	0.82419
RTN3	10313	0.98416
SEPHS2	22928	0.84210
SERP1	27230	0.97835
ST13	6767	0.90375
TMED2	10959	0.92850
VAMP8	8673	0.94863

NOTCH1 unfavorable (lumA high; TN high)

(*Continued*)

lumA_low	lumA_high	TN_low	TN_high	Probe ID	Description	Correlation
				C1S	716	0.77969
				CALD1	800	0.93720
				COL18A1	80781	0.98021
				COL4A1	1282	0.96986
				CTSK	1513	0.94972
				DPYSL3	1809	0.99520
				DSP	1832	0.95909
				FBLN1	2192	0.91591
				FBLN2	2199	0.99000
				GSN	2934	0.98842
				IGFBP7	3490	0.95058
				ITGB1	3688	0.89581
				LAMB1	3912	0.96046
				MMP11	4320	0.89608
				MRC2	9902	0.99240
				NOTCH3	4854	0.86646
				PALLD	23022	0.99306
				PDGFRB	5159	0.91533
				PLEC	5339	0.97589
				PTRF	284119	0.99999
				RPL3	6122	0.81793
				SERPING1	710	0.91245
				SULF1	23213	0.98701
				TAGLN	6876	0.98411
				THBS2	7058	0.93469
				TIMP2	7077	0.98945
				TLN1	7094	0.93785
				ZFP36L1	677	0.99301

NOTCH1 unfavorable (lumA high; TN high)

lumA_low	lumA_high	TN_low	TN_high	Probe ID	Description	Correlation
				IGFBP5	3488	0.94959
				MGP	4256	0.98735
				POSTN	10631	0.88805
				TIMP3	7078	0.87294
				TPT1	7178	0.82300

NOTCH3 favorable (lumA low; TN low)

lumA_low	lumA_high	TN_low	TN_high	Probe ID	Description	Correlation
				B2M	567	0.92443
				CD74	972	0.92463
				HSP90AB1	3326	0.97656
				YWHAZ	7534	0.93857

NOTCH3 favorable (lumA low; TN low)

(*Continued*)

lumA_low	lumA_high	TN_low	TN_high	Probe ID	Description	Correlation
				ATP5A1	498	0.88423
				BCAP31	10134	0.95962
				EIF4A2	1974	0.91744
				HMGN2	3151	0.88160
				HNRNPA2B1	3181	0.88149
				HSPA5	3309	0.94600
				NPM1	4869	0.86037
				PRDX1	5052	0.92354
				RPN2	6185	0.89517
				SERP1	27230	0.94228
				UBB	7314	0.95238
				WBP2	23558	0.97065

NOTCH3 unfavorable (lumA high; TN high)

lumA_low	lumA_high	TN_low	TN_high	Probe ID	Description	Correlation
				COL1A1	1277	0.99772
				COL1A2	1278	0.99248
				COL3A1	1281	0.99324
				FN1	2335	0.99346

NOTCH3 unfavorable (lumA high; TN high)

lumA_low	lumA_high	TN_low	TN_high	Probe ID	Description	Correlation
				CNN2	1265	0.95301
				DDR1	780	0.82591
				FBLN1	2192	0.85388
				HLA-DRB1	3123	0.91566
				LAPTM5	7805	0.96979
				LTF	4057	0.98025
				PDIA3	2923	0.75155
				PTRF	284119	0.99668
				RAC1	5879	0.88635
				SERP1	27230	0.83707
				SERPING1	710	0.93764
				TACSTD2	4070	0.83502
				TPM1	7168	0.92976

ADAM10 favorable (lumA low; TN high)

lumA_low	lumA_high	TN_low	TN_high	Probe ID	Description	Correlation
				B2M	567	1.00000

ADAM10 unfavorable (lumA high; TN low)

(*Continued*)

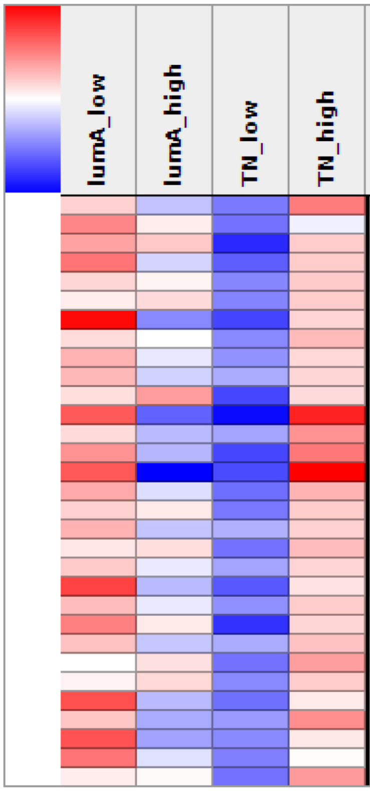

Probe ID	Description	Correlation
AKT1	207	0.91887
ARL6IP1	23204	0.83811
CANX	821	0.89269
CD63	967	0.97663
COL11A1	1301	0.94537
CPD	1362	0.83152
CPT1A	1374	0.89686
CRIP2	1397	0.94623
CYB561	1534	0.99323
CYP51A1	1595	0.97442
DSTN	11034	0.71219
DYNC1H1	1778	0.94935
EGLN3	112399	0.87415
EIF5	1983	0.98290
EPCAM	4072	0.82853
FXYD3	5349	0.99952
GNS	2799	0.92260
HIST1H1C	3006	0.94509
HN1L	90861	0.86616
HNRNPF	3185	0.99986
HSPA9	3313	0.92296
KIAA0182	23199	0.99925
KTN1	3895	0.93972
LPCAT3	10162	0.95544
LRP10	26020	0.80397
NEBL	10529	0.80870
PDLIM5	10611	0.90449
RMND5A	64795	0.89352
SEZ6L2	26470	0.86395
SORL1	6653	0.89682
TOB1	10140	0.89029

PSEN1 favorable (lumA high; TN low)

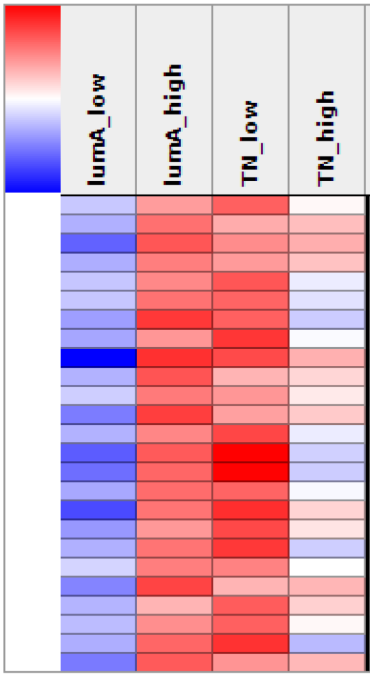

Probe ID	Description	Correlation
APH1A	51107	0.96727
ARHGDIB	397	0.91016
BGN	633	0.92053
CAPNS1	826	0.94889
COPA	1314	0.95777
CTGF	1490	0.95617
CTNNB1	1499	0.95440
H3F3B	3021	0.95623
HSPA1A	3303	0.95079
JUP	3728	0.89813
LAPTM4A	9741	0.98428
LGALS3BP	3959	0.91904
MMP14	4323	0.96578
PTPRF	5792	0.97674
RPL10A	4736	0.96651
RPL15	6138	0.99327
RPL23	9349	0.97521
RPL34	6164	0.97056
RPS14	6208	0.94446
RPS23	6228	0.97702
SPINT2	10653	0.87050
TAPBP	6892	0.92937
TLN1	7094	0.98516
TPM3	7170	0.92920
ZFP36L1	677	0.92599

PSEN1 favorable (lumA high; TN low)

(Continued)

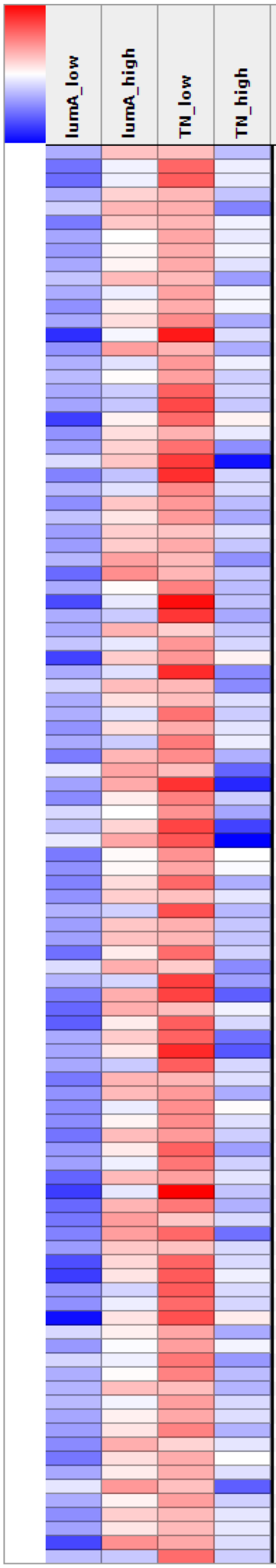

Probe ID	Description	Correlation
AKT2	208	0.92561
ANXA1	301	0.93284
APOE	348	0.93612
ATF6B	1388	0.96754
BRD2	6046	0.82471
BTG1	694	0.88959
C1QA	712	0.96247
C1QB	713	0.94262
C1QC	714	0.98558
C1orf43	25912	0.86139
C6orf106	64771	0.92108
CALD1	800	0.93364
CALM2	805	0.98476
CALU	813	0.94800
CAP1	10487	0.88559
CDC42EP1	11135	0.89468
CDC42SE1	56882	0.98720
CDH3	1001	0.87809
CHI3L1	1116	0.88071
CLIC1	1192	0.88907
CNN2	1265	0.94879
COL18A1	80781	0.96940
COL4A1	1282	0.79875
COTL1	23406	0.88805
CX3CL1	6376	0.92077
CYR61	3491	0.97928
DAP3	7818	0.95649
DGCR2	9993	0.92781
DSP	1832	0.97472
EGR1	1958	0.82707
EIF3A	8661	0.85800
EIF3D	8664	0.98311
EIF3E	3646	0.95554
EIF4G1	1981	0.88320
ENSA	2029	0.85157
EWSR1	2130	0.92442
F11R	50848	0.85603
FBL	2091	0.90876
FKBP10	60681	0.80714
FLOT2	2319	0.97514
FOXP4	116113	0.92732
GALNT2	2590	0.96374
GLT25D1	79709	0.84884
GLTSCR2	29997	0.97105
GNAI2	2771	0.66342
GSN	2934	0.89429
HNRNPA3	220988	0.99390
HNRNPM	4670	0.89364
HNRNPUL1	11100	0.87617
HSPA5	3309	0.73594
IGFBP3	3486	0.90970
ITGA6	3655	0.92216
ITGB4	3691	0.99952
KLF6	1316	0.92118
KRT17	3872	0.88692
LAMB1	3912	0.95989
LAP3	51056	0.94508
LGALS1	3956	0.98513
MAP4	4134	0.71573
MYC	4609	0.89276
NAP1L1	4673	0.95984
NCSTN	23385	0.83483
NFIX	4784	0.97427
NOTCH3	4854	0.93667
NPM1	4869	0.92547
PABPC4	8761	0.87190
PBXIP1	57326	0.89760
PLEC	5339	0.96046
PLS3	5358	0.88310
PTBP1	5725	0.97802
PTRF	284119	0.95456
RPL14	9045	0.98482
RPL18A	6142	0.95855
RPL23A	6147	0.91603
RPL24	6152	0.95412
RPS3A	6189	0.97804
S100A16	140576	0.82277
SET	6418	0.94289
SHC1	6464	0.92758
SLC39A7	7922	0.96959
SLC44A2	57153	0.93954
SLC5A6	8884	0.90451
SMARCA4	6597	0.95475
SND1	27044	0.88206
SPP1	6696	0.90969
SUMO2	6613	0.93680
SUN2	25777	0.87262
SUPT5H	6829	0.97972
TNKS1BP1	85456	0.91035
TPM2	7169	0.96409
TYMP	1890	0.99099
UBAP2L	9898	0.99596
UBE2D3	7323	0.80835
UFC1	51506	0.88988
USF2	7392	0.98790
VAT1	10493	0.65272
VTCN1	79679	0.99741
WASF2	10163	0.92699
WBP2	23558	0.96487
WDR1	9948	0.85649
XRCC6	2547	0.84816

PSEN1 unfavorable (lumA low; TN high)

(*Continued*)

lumA_low	lumA_high	TN_low	TN_high	Probe ID	Description	Correlation
				RPL15	6138	0.97171
				RPL18	6141	0.98847
				RPL27A	6157	0.99260
				RPL28	6158	0.99366
				RPL32	6161	0.99912
				RPL35A	6165	0.97639
				RPL37	6167	0.99280
				RPL3	6122	0.97910
				RPLP0	6175	0.98589
				RPS14	6208	0.97067
				RPS8	6202	0.98056

HES1 favorable (lumA high; TN low)

lumA_low	lumA_high	TN_low	TN_high	Probe ID	Description	Correlation
				CANX	821	0.89421
				CAPRIN1	4076	0.94726
				CTSD	1509	0.83377
				EIF4G2	1982	0.95626
				FBN1	2200	0.93594
				HSPG2	3339	0.92563
				MACF1	23499	0.95646
				SULF1	23213	0.96106
				THBS1	7057	0.97987
				THBS2	7058	0.99313
				TIMP2	7077	0.99377
				VCAN	1462	0.99075

HES1 favorable (lumA high; TN low)

(Continued)

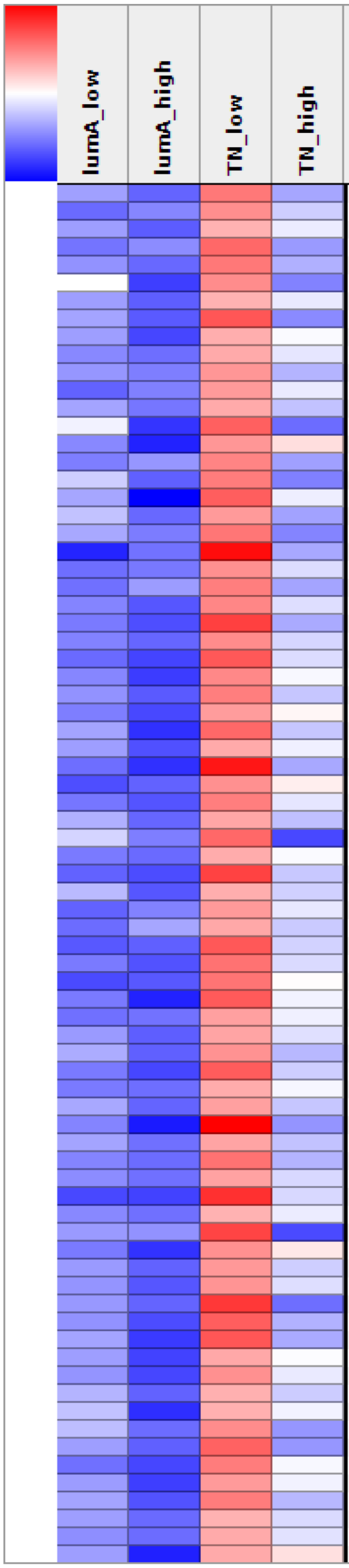

Probe ID	Description	Correlation
ANLN	54443	0.98122
APH1A	51107	0.96431
ARHGAP21	57584	0.97262
ARHGEF2	9181	0.94372
ARPC2	10109	0.99035
ATAD2	29028	0.82182
ATP2C1	27032	0.97562
C6orf106	64771	0.95715
CCT8	10694	0.94804
CDC25B	994	0.98247
CDC42SE1	56882	0.98612
CMPK1	51727	0.94778
CNN3	1266	0.99791
CNOT1	23019	0.85205
CORO1C	23603	0.91309
CXCR4	7852	0.93992
DNMT1	1786	0.90344
DSG2	1829	0.96041
ENAH	55740	0.94993
ETS1	2113	0.92825
F11R	50848	0.94073
FDPS	2224	0.97503
FURIN	5045	0.92940
GARS	2617	0.99445
GLG1	2734	0.98957
GLS	2744	0.99558
GLT25D1	79709	0.99458
GNB4	59345	0.97411
HIF1A	3091	0.99956
HMGB2	3148	0.94765
HNRNPA2B1	3181	0.98768
HNRNPUL1	11100	0.96836
HYOU1	10525	0.99272
ITM2C	81618	0.91680
IVNS1ABP	10625	0.98828
LAMB1	3912	0.98819
LAMP2	3920	0.79708
LBR	3930	0.95254
LCP1	3936	0.99330
LTBP1	4052	0.97046
MAN2B1	4125	0.94722
MCAM	4162	0.91916
MMP9	4318	0.98125
MYBL2	4605	0.99611
NASP	4678	0.94810
NCAPD2	9918	0.98651
NOTCH1	4851	0.95970
PCNA	5111	0.98918
PLOD2	5352	0.98866
PLS3	5358	0.99933
PODXL	5420	0.95651
PRC1	9055	0.99513
PSMD2	5708	0.98499
QKI	9444	0.99749
SDCBP	6386	0.98968
SEC63	11231	0.99468
SLC25A5	292	0.98676
SLC2A1	6513	0.97194
SLC39A7	7922	0.84204
SLC5A6	8884	0.93519
SMARCA4	6597	0.99880
SMC4	10051	0.99229
SMG5	23381	0.93195
SRPK1	6732	0.99337
SSR1	6745	0.98334
STARD7	56910	0.94878
STIP1	10963	0.98284
TMPO	7112	0.98412
TUG1	55000	0.90811
UBAP2L	9898	0.94267
USP1	7398	0.96961
WARS	7453	0.97573
XPO1	7514	0.96689
XPO5	57510	0.99136
YARS	8565	0.99280
YEATS2	55689	0.98805
YWHAG	7532	0.88798

HES1 unfavorable (lumA low; TN high)

(*Continued*)

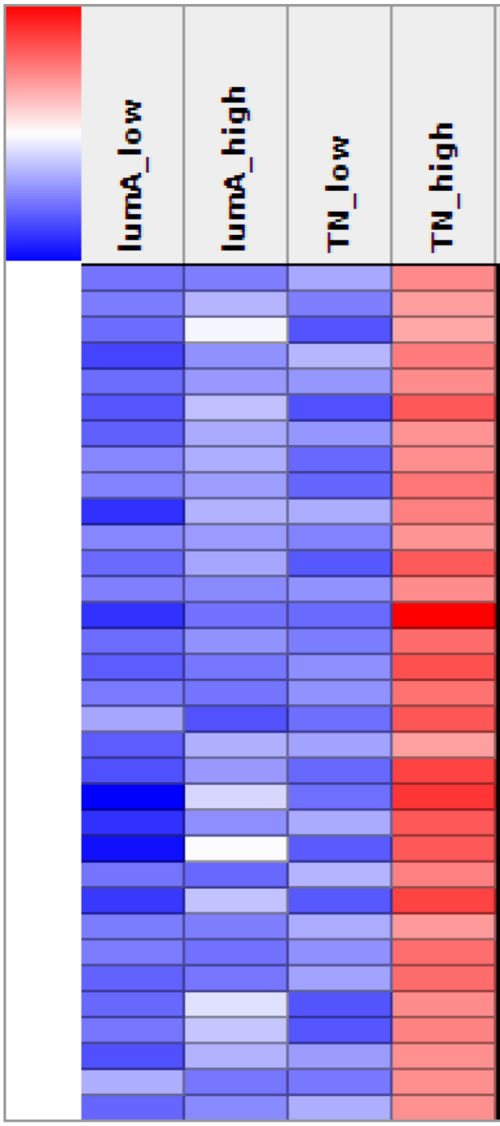

Probe ID	Description	Correlation
A2M	2	0.97496
APOE	348	0.99173
CD151	977	0.87606
CLIC1	1192	0.96613
CNN2	1265	0.99678
COL18A1	80781	0.97833
FKBP8	23770	0.99482
GPX1	2876	0.97835
GRN	2896	0.98332
GSN	2934	0.95717
ITGA5	3678	0.99194
JUN	3725	0.98781
LAMB3	3914	0.98817
LGALS1	3956	0.99731
LTF	4057	0.99739
MMP9	4318	0.98865
MT2A	4502	0.97737
PDIA3	2923	0.91998
PMEPA1	56937	0.98401
PTRF	284119	0.99896
RPL18	6141	0.95873
RPL24	6152	0.97459
RPL28	6158	0.91815
RRBP1	6238	0.94495
S100A16	140576	0.98312
S100A9	6280	0.96619
SAT1	6303	0.97585
SH3BGRL3	83442	0.97759
TAGLN	6876	0.93386
THY1	7070	0.96079
UBB	7314	0.98418
ZFP36L1	677	0.92249
ZYX	7791	0.97751

LFNG favorable (lumA high; TN low)

(Continued)

Probe ID	Description	Correlation
ACSL3	2181	0.94635
ADNP	23394	0.99448
ANKRD17	26057	0.97849
APLP2	334	0.98133
ARFGEF1	10565	0.93266
ARL6IP1	23204	0.97984
ASH1L	55870	0.98322
AZIN1	51582	0.97050
CAND1	55832	0.98074
CAPRIN1	4076	0.97844
CCNI	10983	0.93060
CDH1	999	0.97603
CTNND1	1500	0.99232
DCAF7	10238	0.99510
DDB1	1642	0.97550
DDX17	10521	0.92912
DDX3X	1654	0.94978
EIF4A2	1974	0.99717
GFPT1	2673	0.96628
GOLGA4	2803	0.98225
GTF2I	2969	0.95959
HNRNPK	3190	0.97371
HSPA9	3313	0.86993
IPO7	10527	0.94327
IQGAP1	8826	0.99894
ITPR2	3709	0.97811
KIF5B	3799	0.93956
LMAN1	3998	0.97334
MACF1	23499	0.92984
MAL2	114569	0.99468
NOTCH2	4853	0.95702
NUP210	23225	0.94878
RAD23B	5887	0.98234
RTN3	10313	0.99889
SF3B1	23451	0.99951
SMARCA2	6595	0.95515
SMG1	23049	0.98125
SON	6651	0.93233
SPINT2	10653	0.72367
STAT3	6774	0.98133
TBL1XR1	79718	0.95451
TM9SF3	56889	0.99604
TMED2	10959	0.93059
UBR5	51366	0.93623
WNK1	65125	0.99825
XRCC5	7520	0.99364
ZNF664	144348	0.88859

LFNG favorable (lumA high; TN low)

Probe ID	Description	Correlation
CD24	100133941	0.99851
EEF1A1	1915	0.90512
PABPC1	26986	0.97312

LFNG unfavorable (lumA low; TN high)

Probe ID	Description	Correlation
ALDOA	226	0.98273
CD74	972	0.89954
SPARC	6678	0.92054

LFNG unfavorable (lumA low; TN high)

Supplementary File 8: Heatmaps of common expression profiles of Notch downstream targets.
