# Supplementary material for: Common profiles of Notch signaling differentiate disease-free survival in luminal type A and triple negative breast cancer
Source: Oncotarget. 2016 Nov 19;8(4):6013–32. doi: 10.18632/oncotarget.13451 (PMC5351609; doi:10.18632/oncotarget.13451)
Supplement: Supplementary file 2 [file oncotarget-08-6013-s002.docx]

**Table 1. Uni- and multivariate Cox analyses for lumA BC.**

|  | Univariate analysis | | | | Multivariate analysis | | | |
| --- | --- | --- | --- | --- | --- | --- | --- | --- |
|  | Coefficient (coef) | HR [exp (coef)] | 95% CI | P-value | Coefficient (coef) | HR [exp (coef)] | 95% CI | P-value |
|  |  | | | | | | | |
| age | 0.0136 | 0.9864 | 0.9439 - 1.031 | 0.54 | -9.98e-01 | 3.69e-01 | 6.618e-43 - 2.053e+41 | 0.98 |
|  |  | | | | | | | |
| lymph nodes | 1.2406 | 0.2892 | 0.0332 - 2.519 | 0.26 | -3.57e+01 | 3.22e-16 | 0.000e+00 - Inf | 0.95 |
| Stage |  | | | | | | | |
| stage ia | -1.84e+01 | 1.05e-08 | 0.00000 - Inf | 0.9991 | 2.56e+01 | 1.34e+11 | 0.000e+00 - Inf | 0.99 |
| stage ib | -1.88e+01 | 7.12e-09 | 0.00000 - Inf | 0.9999 | 0.00e+00 | 1.00e+00 | 1.000e+00 - 1.000e+00 | NA |
| stage ii | NA | NA | NA | NA | 0.00e+00 | 1.00e+00 | 1.000e+00 - 1.000e+01 | NA |
| stage iia | -5.44e-02 | 9.47e-01 | 0.08553 - 10.49 | 0.9646 | 7.14e+00 | 1.26e+03 | 0.000e+00 - Inf | 0.99 |
| stage iib | 1.07e+00 | 2.90e+00 | 0.33727 - 24.96 | 0.3320 | 5.92e+00 | 3.72e+02 | 0.000e+00 - Inf | 0.99 |
| stage iiia | -1.77e+01 | 1.97e-08 | 0.00000 - Inf | 0.9978 | -5.72e+01 | 1.49e-25 | 0.000e+00 - Inf | 0.97 |
| stage iiib | 2.19e+00 | 8.92e+00 | 0.54245 - 146.65 | 0.1256 | 4.69e+01 | 2.37e+20 | 0.000e+00 - Inf | 1.00 |
| stage iiic | 2.06e+00 | 7.87e+00 | 0.68228 - 90.72 | 0.0982 | 3.73e+01 | 1.56e+16 | 0.000e+00 - Inf | 0.98 |
| stage iv | 3.88e+00 | 4.83e+01 | 4.23417 - 551.00 | 0.0018 | 4.69e+01 | 2.45e+20 | 0.000e+00 - Inf | 0.98 |
| stage x | 1.65e+00 | 5.21e+00 | 0.31341 - 86.57 | 0.2498 | 3.19e+01 | 7.20e+13 | 0.000e+00 - Inf | 0.99 |
| Histology |  | | | | | | | |
| histological_typeinfiltrating lobular carcinoma | -5.63e-01 | 5.69e-01 | 0.1546 - 2.096 | 0.4 | -2.20e+01 | 2.71e-10 | 1.348e-312 - 5.462e+292 | 0.95 |
| histological_typemetaplastic carcinoma | NA | NA | NA | NA | 0.00e+00 | 1.00e+00 | 1.000e+00 - 1.000e+00 | NA |
| histological_typemixed histology (please specify) | -1.83e+01 | 1.13e-08 | 0.0000 - Inf | 1.0 | 3.98e+01 | 1.94e+17 | 0.000e+00 - Inf | 0.98 |
| histological_typemucinous carcinoma | -1.83e+01 | 1.16e-08 | 0.0000 - Inf | 1.0 | 8.18e+01 | 3.20e+35 | 3.201e+35 - 3.201e+35 | 2,00E-16 |
| histological_typeother specify | -1.83e+01 | 1.10e-08 | 0.0000 - Inf | 1.0 | -1.95e+01 | 3.53e-09 | 0.000e+00 - Inf | 0.97 |
| Menopause status |  | | | | | | | |
| menopause_statusperi (6-12 months since last menstrual period) | -1.78e+01 | 1.85e-08 | 0.0000 - Inf | 0.998 | -4.33e+00 | 1.32e-02 | 0.000e+00 - Inf | 1.00 |
| menopause_statuspost (prior bilateral ovariectomy or >12 mo since lmp with no prior hysterectomy) | -9.78e-01 | 3.76e-01 | 0.1295 - 1.092 | 0.072 | 3.40e+01 | 5.92e+14 | 0.000e+00 - Inf | 0.97 |
| menopause_statuspre (<6 months since lmp and no prior bilateral ovariectomy and not on estrogen replacement) | NA | NA | NA | NA | 0.00e+00 | 1.00e+00 | 1.000e+00 - 1.000e+00 | NA |
| Genes |  | | | | | | | |
| ADAM17 | 0.00185 | 1.00185 | 0.9994 - 1.004 | 0.13 | 1.56e-01 | 1.17e+00 | 1.932e-03 - 7.070e+02 | 0.96 |
| DLL4 | -0.00194 | 0.99806 | 0.994 - 1.002 | 0.35 | -8.93e-02 | 9.15e-01 | 1.906e-02 - 4.388e+01 | 0.96 |
| HES1 | -0.000669 | 0.999331 | 0.9985 - 1 | 0.12 | -5.90e-03 | 9.94e-01 | 3.312e-01 - 2.984e+00 | 0.99 |
| HES4 | -0.004708 | 0.995303 | 0.9867 - 1.004 | 0.29 | -2.37e-06 | 1.00e+00 | 9.962e-01 - 1.004e+00 | 1.00 |
| HES5 | -2.48e-07 | 1.00e+00 | 1 | 0.47 | 1.11e-05 | 1.00e+00 | 9.995e-01 - 1.001e+00 | 0.97 |
| HEY1 | 0.000459 | 1.000459 | 0.9995 - 1.001 | 0.32 | 2.19e-02 | 1.02e+00 | 3.156e-01 - 3.311e+00 | 0.97 |
| JAG2 | -0.000763 | 0.999237 | 0.9975 - 1.001 | 0.4 | -2.40e-02 | 9.76e-01 | 1.231e-01 - 7.744e+00 | 0.98 |
| LFNG | -6.49e-05 | 1.00e+00 | 0.9994 - 1 | 0.81 | 1.68e-02 | 1.02e+00 | 5.621e-01 - 1.840e+00 | 0.96 |
| NOTCH1 | 0.000172 | 1.000172 | 0.9991 - 1.001 | 0.75 | -3.20e-03 | 9.97e-01 | 2.497e-01 - 3.979e+00 | 1.00 |
| NOTCH3 | 0.000101 | 1.000101 | 0.9999 - 1 | 0.33 | 7.08e-03 | 1.01e+00 | 8.574e-01 - 1.183e+00 | 0.93 |
| NUMB | -0.001105 | 0.998896 | 0.9974 - 1 | 0.15 | 5.39e-03 | 1.01e+00 | 4.183e-01 - 2.417e+00 | 0.99 |
| ADAM10 | 0.000372 | 1.000372 | 0.9996 - 1.001 | 0.35 | -1.86e-02 | 9.82e-01 | 4.898e-02 - 1.967e+01 | 0.99 |
| PSEN1 | -0.000784 | 0.999216 | 0.9981 - 1 | 0.16 | -3.38e-02 | 9.67e-01 | 9.468e-02 - 9.870e+00 | 0.98 |
