# Supplementary material for: Common profiles of Notch signaling differentiate disease-free survival in luminal type A and triple negative breast cancer
Source: Oncotarget. 2016 Nov 19;8(4):6013–32. doi: 10.18632/oncotarget.13451 (PMC5351609; doi:10.18632/oncotarget.13451)
Supplement: Supplementary file 3 [file oncotarget-08-6013-s003.docx]

**Table 2. Uni- and multivariate Cox analyses for TN BC.**

|  | Univariate analysis | | | | Multivariate analysis | | | |
| --- | --- | --- | --- | --- | --- | --- | --- | --- |
|  | Coefficient (coef) | HR [exp (coef)] | 95% CI | P-value | Coefficient (coef) | HR [exp (coef)] | 95% CI | P-value |
|  |  | | | | | | | |
| age | 0.0039 | 1.0039 | 0.9026 - 1.117 | 0.94 | NA | NA | NA | NA |
|  |  | | | | | | | |
| lymph nodes | NA | NA | NA | NA | NA | NA | NA | NA |
| Stage |  | | | | | | | |
| stagestage ia | 2.15e+01 | 2.21e+09 | 0 - Inf | 1 | 3.90e+01 | 8.68e+16 | 0 - Inf | 1 |
| stagestage iia | -2.10e+01 | 7.69e-10 | 0 - Inf | 1 | 1.77e+01 | 4.90e+07 | 0 - Inf | 1 |
| stagestage iib | -2.11e+01 | 7.13e-10 | 0 - Inf | 1 | 8.57e+00 | 5.29e+03 | 0 - Inf | 1 |
| stagestage iiia | -1.81e+00 | 1.64e-01 | 0 - Inf | 1 | -4.06e+01 | 2.41e-18 | 0 - Inf | 1 |
| stagestage iiib | 1.75e+02 | 6.83e+75 | 0 - Inf | 1 | 3.68e+02 | 9.09e+159 | 9.090e+159 - 9.090e+159 | <2e-16 |
| stagestage iiic | -1.24e+01 | 4.32e-06 | 0 - Inf | 1 | 0 | 1 | 1 | NA |
| stagestage iv | 5.65e+01 | 3.52e+24 | 0 - Inf | 1 | 0 | 1 | 1 | NA |
| Histology |  | | | | | | | |
| histologyinfiltrating lobular carcinoma | -2.56e+01 | 7.71e-12 | 0 - Inf | 1 | 1.42e+02 | 5.42e+61 | 0 - Inf | 1 |
| histologymedullary carcinoma | NA | NA | NA | NA | -2.59e+00 | 7.49e-02 | 7.487e-02 - 7.487e-02 | <2e-16 |
| histologymetaplastic carcinoma | NA | NA | NA | NA | -5.16e+01 | 3.98e-23 | 0 - Inf | 1 |
| histologymixed histology (please specify) | 3.79e+01 | 2.98e+16 | 0 - Inf | 1 | 8.13e+01 | 2.11e+35 | 0 - Inf | 1 |
| histologyother specify | -1.90e+01 | 5.67e-09 | 0 - Inf | 1 | 4.32e+00 | 7.48e+01 | 0 - Inf | 1 |
| Menopause status |  | | | | | | | |
| menopauseperi (6-12 months since last menstrual period) | NA | NA | NA | NA | NA | NA | NA | NA |
| menopausepost (prior bilateral ovariectomy or >12 mo since lmp with no prior hysterectomy) | -1.76e+01 | 2.30e-08 | 0 - Inf | 1 | NA | NA | NA | NA |
| menopausepre (<6 months since lmp and no prior bilateral ovariectomy and not on estrogen replacement) | NA | NA | NA | NA | NA | NA | NA | NA |
| Genes |  | | | | | | | |
| HES1 | -9.49e-05 | 1 | 0.9986 - 1.001 | 0.89 | 1.14e-02 | 1.01e+00 | 0 - Inf | 1 |
| LFNG | -0.00196 | 0.99804 | 0.9909 - 1.005 | 0.59 | -1.78e-02 | 9.82e-01 | 0 - Inf | 1 |
| NOTCH1 | 0.000593 | 1.000593 | 1 - 1.001 | 0.025 | 5.32e-03 | 1.01e+00 | 2.941e-49 - 3.437e+48 | 1 |
| NOTCH2 | -3.89e-05 | 1 | 0.9997 - 1 | 0.75 | 7.34e-04 | 1 | 2.966e-40 - 3.377e+39 | 1 |
| NOTCH3 | 8.65e-05 | 1 | 1 | 0.18 | -1.04e-03 | 9.99e-01 | 3.447e-33 - 2.895e+32 | 1 |
| ADAM10 | -0.00229 | 0.99771 | 0.9946 - 1.001 | 0.15 | 3.71e-03 | 1 | 3.575e-132 - 2.818e+131 | 1 |
| PSEN1 | 9.18e-05 | 1 | 0.9987 - 1.002 | 0.9 | -3.76e-07 | 1 | 2.727e-239 - 3.667e+238 | 1 |
